# Mitochondrial-Targeted SS-31 Attenuates the Doxorubicin-Induced Cardiomyoblast H9C2 Cell Senescence

**DOI:** 10.3390/biology15131034

**Published:** 2026-06-28

**Authors:** Jiaojiao Fan, Jinzi Wu, Shuo Yan, Songlin Li, Peter S. Rabinovitch, Xingyun Qi, Huiliang Zhang

**Affiliations:** 1 Department of Pharmacology and Toxicology, University of Arkansas for Medical Sciences, 4301 West Markham Street, Little Rock, AR 72205, USA; 2 Department of Medical Genetics & Molecular Biochemistry, Lewis Katz School of Medicine, Temple University, 3440 N Broad St., Kresge Hall, Philadelphia, PA 19140, USA; 3 Department of Laboratory Medicine and Pathology, University of Washington, 1959 NE Pacific St., Seattle, WA 98195, USA; 4 JHSC Building, Department of Biology, Rutgers University, Camden, NJ 08103, USA

**Keywords:** doxorubicin, H9C2 cell, senescence, mitochondria, SS-31

## Abstract

Doxorubicin (DOX), an effective chemotherapeutic agent for many types of cancer, is known for significant cardiotoxic side effects, which largely limit its clinical usage. A 3 h treatment of cardiomyoblast H9C2 cells with a low concentration of DOX (100 nM) can induce senescence-associated β-galactosidase (SA β-gal) staining, a gold standard of cell senescence. In the current study, we comprehensively characterized the phenotype of the DOX-induced senescent cardiomyocytes for the first time. Establishing this *in vitro* model will facilitate an expanded capacity for searching for effective treatments for DOX-induced cell senescence. Using SA β-gal staining and cell growth rate as readouts, we assessed the concentration-dependent effect of DOX on H9C2 cell senescence. The cells were treated with DOX for 3 h and subsequently cultured for 3 days. We found that a 50 nM concentration of DOX induced ~50% SA β-gal staining and completely inhibited cell growth. The DOX-induced H9C2 cell senescence was further confirmed by several well-accepted senescence markers, including cell hypertrophy, increased p16 and p21 expression, increased Senescence Associated Secretory Phenotype (SASP) markers, arrested cell cycle, and increased ROS production. Interestingly, we found that 50 nM DOX increased mitochondrial respiration. Translationally, we found that mitochondrial-targeted tetrapeptide SS-31 (elamipretide, 1 µM) partially attenuated 50 nM DOX-induced SA β-gal staining from 51.4% to 35.8%. SS-31 also prevented increases in the p16, p21, and SASP markers and mitigated mitochondrial ROS production. Additionally, SS-31 reversed the 50 nM DOX-induced elevation of mitochondrial respiration. However, 1 µM SS-31 failed to prevent the cell cycle arrest induced by 50 nM DOX. Using a 3 h treatment of 50 nM DOX, we established an H9C2 cell senescence model. Treatment with SS-31 attenuates this DOX-induced cell senescence but not the cell cycle arrest. These data suggest that SS-31 is a promising drug to treat DOX-induced cardiomyocyte senescence.

## Simple Summary

Doxorubicin is a powerful cancer drug, but its clinical use is heavily restricted by severe heart-damaging side effects, such as cellular senescence. To address this problem, this study aimed to create a reliable cell model to investigate how this aging process occurs and to test potential treatments. By exposing H9C2 cardiomyoblast cells to a low dose of doxorubicin, we successfully established a senescence model characterized by distinct aging markers and mitochondrial dysfunction. Furthermore, we demonstrated that a protective compound, SS-31, effectively reduced the H9C2 cells from doxorubicin-induced damage by reversing key signs of aging and reducing cellular stress. This research provides a valuable tool for screening future heart-protective therapies and highlights SS-31 as a promising strategy to prevent chemotherapy-related heart damage.

## 1. Introduction

Doxorubicin (DOX) is one of the most effective broad-spectrum anti-cancer chemotherapy agents, utilized either as a monotherapy or in combination with other drugs or radiotherapy to battle cancer. Despite its efficacy, DOX treatment is limited by substantial cardiotoxicity, premature cardiac aging, and heart failure during active treatment or in a long-term post-chemotherapy [1]. However, there are no therapeutic interventions to prevent or reverse DOX-induced cardiac dysfunction without interfering with its anti-neoplastic efficacy.

Multiple mechanisms of DOX-induced myocyte injury have been proposed and extensively investigated in past decades, including nuclear DNA damage, oxidative stress, apoptosis [2], and mitochondrial dysfunction [3]. In particular, mitochondrial injury has garnered increasing attention in recent years as a crucial pathogenic event contributing to DOX-induced cardiac toxicity [3]. Another major cardiotoxic profile of DOX is dose-dependent; high doses of DOX typically cause acute cardiac toxicity, whereas lower doses may trigger cardiac senescence [4]. In this regard, there is growing recognition that cardiac cell senescence, characterized by homeostatic imbalance and loss of function, contributes to many cardiac disorders and leads to age-related cardiac failure [5,6,7,8].

Preclinical studies have demonstrated that a single dose of DOX (20 mg/kg, i.p.) causes acute cardiac dysfunction in mice by 5 days [9], whereas repeated (4–6) low-dose injections (2.5 mg/kg i.p.) induce cardiac senescence in mice and rats by four and eleven months, respectively [10,11,12]. Similar trends are observed *in vitro*, where micromolar concentrations of DOX induce acute cell toxicity in H9C2 cardiomyocytes by 24 h [13], while a lower nanomolar concentration of DOX (100 nM) induces senescence by 7 days in both H9C2 cells [13,14] and in neonatal cardiomyocytes [13]. Moreover, recent reports showed that even 50 nM DOX induces senescence in human induced pluripotent stem differentiated cardiomyocytes (hiPSC-CMs) for 4 days [15,16] and neonatal rat cardiomyocytes for 3 days [15]. Importantly, clinical observations have confirmed the presence of DOX-induced senescence in human cardiomyocytes. A recent study demonstrated that biopsies from the left ventricles of patients with DOX-induced cardiotoxicity exhibited increased senescence-related markers compared to biopsies from control patients, whereas apoptotic pathways were not activated in the same biopsies [17]. Furthermore, the same authors reported that dynamically engineered heart tissues exposed to multiple low concentrations of DOX to mimic clinical treatment scenarios also expressed increased senescence markers that positively correlated with tissue dilatation, decreased force generation, and troponin release [17].

Considering the growing evidence that senescence is a feature of DOX-induced cardiac dysfunction [18], there is a critical need for validated cardiomyocyte models suitable for high-throughput drug screening to identify small molecules with anti-senescence properties [19]. Accordingly, the goal of the present study was to provide a comprehensive characterization of a DOX-induced H9C2 cardiomyocyte senescence model. This model builds up a single 3 h treatment with a lower concentration of DOX (50 nM), which induces H9C2 cell senescence within three days. Subsequently, we utilized the validated H9C2 cell senescence model for initial drug testing to determine whether the tetrapeptide SS-31 (elamipretide) can attenuate DOX-induced H9C2 senescence. SS-31 targets the mitochondrial inner membrane by interacting with cardiolipin [20], thereby stabilizing cristae curvature, preventing cytochrome C oxidative modification and release [21], and reducing reactive oxygen species (ROS) production [22]. It confers protection to many organs in a broad spectrum of animal disease models, including heart failure [23,24,25,26]. SS-31 protects against one dose of 20 mg/kg DOX-induced heart dysfunction in the rat [27] through the prevention of DOX-induced proteolytic signaling [28]. SS-31 mitigates excessive mitochondrial proton leak [29], improves cardiac function in aged mice [30], and attenuates acute toxicity in H9C2 cells caused by high DOX concentrations [31]. In this present study, focused on the DOX-induced senescence, we evaluate the anti-senescence potential of SS-31 as a proof-of-concept for drug screening within our DOX-induced H9C2 cell senescence model.

## 2. Materials and Methods

### 2.1. Cell Culture and Treatment

**Figure 1:**
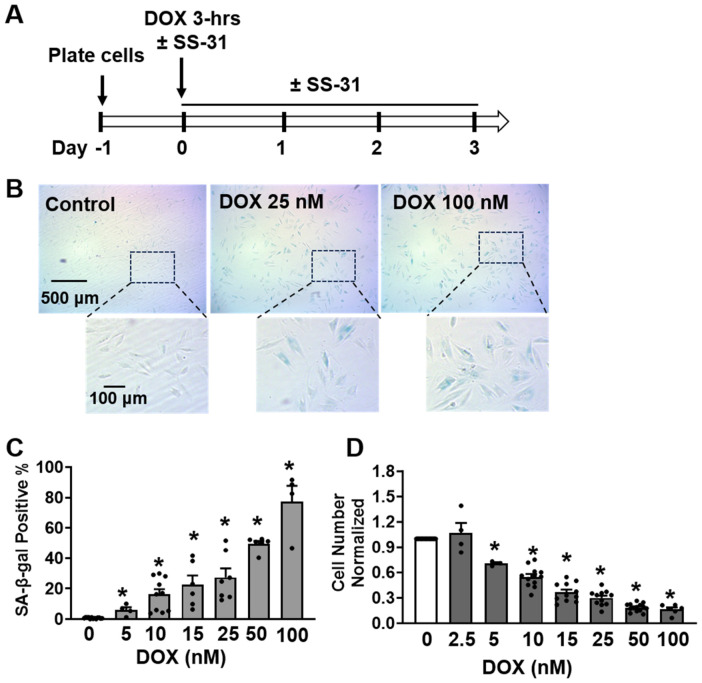
Low-concentration doxorubicin (DOX) induces a senescent phenotype in H9C2 cells. (**A**) Schematic overview of the experiment protocol. H9C2 cells were plated on Day −1. On Day 0, cells were treated with DOX (5–100 nM) with or without SS-31 for 3 h. Following DOX exposure, the medium was replaced with fresh DOX-free medium (with or without SS-31), and cells were maintained in culture for an additional 3 days. With this treatment regimen, SS-31 was added both at hour 0 with DOX and after DOX washout. (**B**) Representative images of senescence-associated β-galactosidase (SA-β-gal) staining. The senesecent cells were stained blue color for positive SA-β-gal activity. Scale bar: 500 μm in the upper panel, and 100 μm in the lower enlarged panel. (**C**) DOX concentrations below 100 nM induced SA-β-gal activity in a dose-dependent manner. N = 4–10. (**D**) Impact of low-dose DOX on cell proliferation. DOX treatment resulted in significant, dose-dependent cell growth retardation. In (**C**,**D**), each black dot represents a single independent experiment. Open bar, no treatment control; Grey bar, DOX added as indicated concentration. N = 3–14. All the data are mean ± SEM, * *p* < 0.01 vs. control.

H9C2 cells were cultured in DMEM (Gibco, Grand Island, NY, USA, Cat# 11965-092) supplemented with 10% fetal bovine serum (Gibco, 15140-122) and 1% penicillin/streptomycin (Gibco, 16140-071) in 5% CO_2_ at 37 °C. To induce senescence, 10^5^ H9C2 cells were plated on a 70 mm diameter dish overnight, and were treated with doxorubicin (DOX, formula C_27_H_29_NO_11_HCl, molecular weight 579.99 g/mol, Tocris Bioscience, Bristol, UK; Cat# 2252) at the indicated concentrations for 3 h as previously reported [13,14]. Then the DOX-containing medium was replaced with fresh DOX-free medium, and the cells were cultured for an additional 3 days (Figure 1A). SS-31 (Structure D-Arg-Dmt-Lys-Phe-NH_2_, Dmt represents 2′,6′-dimethyl-L-tyrosine, formula C_32_H_49_N_9_O_5_, molecular weight 639.8 g/mol, NovoPro, Princeton, NJ, USA; Cat# 318879) was co-administered with DOX and maintained after DOX treatment as indicated in Figure 1A. With this treatment regimen, SS-31 was added both at hour 0 with DOX and after DOX washout. On Day 3, cells were trypsinized with 0.05% trypsin-EDTA (Gibco, Cat# 25200-056) and centrifuged at 300× *g* for 3 min, and resuspended in fresh media and counted using a hemocytometer.

### 2.2. Senescence-Associated β-Galactosidase (SA-β-Gal) Staining

On Day 3 post-treatment, as indicated in Figure 1A, 5 × 10^4^ cells were seeded in a 35 mm dish and allowed to adhere overnight. Cellular senescence was assessed by the gold standard senescence-associated beta-galactosidase (SA-β-Gal) staining kit (CST, Danvers, MA, USA; Cat# 9860). Briefly, cells were fixed in 4% paraformaldehyde for 10 min and washed twice with PBS. The cells were then incubated with the SA-β-gal staining solution (pH 6.0) overnight at 37 °C in a non-CO_2_ incubator. Images were taken by a Microscope Digital Camera (AmScope, Irvine, CA, USA) and analyzed using ImageJ software (Version 1.54i).

### 2.3. Cell Cycle Analysis by Flow Cytometry

EdU (iFluor 488, Abcam, Cambridge, UK; Cat# AB219801) was incubated with the H9C2 cell at a final concentration of 10 μM for at cell culture incubator for 3 h. Cells were trypsinized, washed with ice-cold PBS, and fixed in cold 70% ethanol overnight at 4 °C. The fixed cells were washed with cold PBS twice, followed by incubation with 100 μL of 100 μg/mL RNase A (Thermo Scientific, Waltham, MA, USA; Cat# EN0531) for 30 min at 37 °C. Then, the fixed cells were stained with 50 μg/mL propidium iodide (PI) for 30 min at 4 °C in the dark. The cell cycle was evaluated by flow cytometry (BD LSRFortessa, San Jose, CA, USA) with an excitation of 488 for EdU and 535 nm for PI. Data were analyzed by FlowJo software (Version 10.10.0).

### 2.4. Mitochondrial Respiration Assay

Mitochondrial respiration was quantified using a Seahorse XFe96 Flux Analyzer (Agilent, Santa Clara, CA, USA). H9C2 cells were replated at 5000 cells/well in an XFe96 plate and incubated in 5% CO_2_ at 37 °C for 6 h to allow for cell attachment. The oxygen consumption rate (OCR) in the intact H9C2 cell was measured using the mitochondrial stress assay as reported earlier [32]. In brief, the culture medium was changed to 180 μL of XF DMEM Medium (Agilent, 103575-100) containing 10 mM glucose, 1 mM pyruvate, and 2 mM glutamine, and then incubated for 1 h at 37 °C without CO_2_. During the assay, oligomycin A (5 µM, complex V inhibitor), carbonylcyanide-p-trifluoromethoxyphenylhydrazone (FCCP, 10 µM, mitochondrial uncoupler), and a combination of antimycin A (5 µM, complex III inhibitor) and rotenone (5 µM, complex I inhibitor) were added sequentially. ATP production, mitochondrial proton leak, and mitochondrial respiration control ratio (RCR) were calculated as previously described [33].

### 2.5. Mitochondrial Superoxide and Mitochondrial Membrane Potential Assay

Cells obtained from the protocol in Figure 1A were plated onto a coverslip and allowed to adhere overnight. Cells were subsequently incubated with MitoSOX Red (5 µM) and MitoTracker Green (200 nM) for 30 min at 37 °C, then imaged using an LSM 880 confocal microscope (ZEISS, Oberkochen, Germany). The ratio of MitoSOX Red (excitation wavelength 561 nm, emission wavelength > 565 nm) to MitoTracker Green (excitation wavelength 488 nm, emission wavelength 505–530 nm) was used to determine the mitochondrial superoxide level, as previously established [29]. For the mitochondrial membrane potential assay, cells were incubated with JC-1 (5 µM) for 30 min at 37 °C, then imaged using a Leica SP5 confocal microscope (Leica Microsystems, Wetzlar, Germany), with excitation 488 and emission of 505–530 nm for green monomer, and excitation 488 and emission of >560 nm for red aggregate.

### 2.6. Western Blot

Cells were lysed with iced RIPA (Sigma, St. Louis, MO, USA; Cat# R0278), and the protein concentrations were quantified by a BCA protein assay (ThermoFisher, Hampton, NH, USA; Cat# 23225). After size separation by SDS-PAGE, proteins were transferred onto a PVDF membrane (ThermoFisher, Cat# IPVH00010). The total protein level was determined by the reversible protein stain kit (ThermoFisher, 24585) to serve as a normalization control. Primary antibodies were p16 (ThermoFisher, Cat# PA5-20379, 1:2000), p21 (ThermoFisher, 14-6715-81, 1:4000), TNF-α (Sigma, Cat# AB1837P, 1:3000), IL-1β (Sigma, Cat# AB1832P, 1:2000), IL-6 (Sigma, Cat# SAB5700632, 1:2000), MMP3 (Cell Signaling Technology, Danvers, MA, USA; Cat# 14351, 1:1000), Lamin B1 (Cell Signaling Techonology, Cat# 13435, 1:1000), Citrate synthase (Santa Cruz, Dallas, TX, USA; Cat# sc-390693, 1:1000), γ-H2AX (Abcam, Cat# ab81299), H2AX (Abcam, Cat# ab322655), and OxPhos antibody cocktail (Invitrogen, Carlsbad, CA, USA; Cat# 458099, 1:1000).

### 2.7. Cell ATP Level Assay

Following the DOX treatment, H9C2 cells were plated into opaque 96-well plates at a density of 5000 cells/well and allowed to adhere for 8 h at 37 °C with 5% CO_2_. Parallel wells containing only DMEM were included to serve as background controls. ATP levels were quantified using the CellTiter-Glo^®^ Luminescent Assay (Promega, Madison, WI, USA; Cat# G7573) according to the manufacturer’s instructions. Briefly, 100 μL of CellTiterGlo reagent was added to each well, mixed for 2 min, and incubated for 10 min at room temperature. Luminescence was recorded using a plate reader (Molecular Devices, SpectraMax i3, San Jose, CA, USA).

### 2.8. Flow Cytometric FITC Annexin V Apoptosis Detection

Apoptosis was quantified using the FITC Annexin V Apoptosis Detection Kit I (Santa Cruz, sc-4252 AK) according to the manufacturer’s instructions. In brief, after the DOX treatment, H9C2 cells were washed twice with ice-cold PBS and resuspended in Annexin V binding buffer at a density of 10^5^ cells per 100 μL. The cell suspension was incubated with 5 μL of FITC Annexin V and 5 μL of PI for 15 min at room temperature in the dark. Following the addition of 400 μL of binding buffer, samples were analyzed by flow cytometry (BD LSRFortessa). Unstained, Annexin V-only, and PI-only stained controls were utilized for compensation and quadrant gating.

### 2.9. Statistical Analysis

All data are presented as mean ± SEM. The data were analyzed by one-way ANOVA followed by Dunnett post hoc test or two-tailed Student’s *t*-test with GraphPad Prism 11 (GraphPad Software, San Diego, CA, USA). The differences were considered statistically significant when *p* < 0.05.

## 3. Results

### 3.1. Doxorubicin (DOX) Concentration-Dependently Induces H9C2 Cell Senescence

H9C2 cardiomyocytes were exposed to nanomolar concentrations of DOX, and the senescent phenotype was evaluated via the gold-standard measurements of β-galactosidase (SA-β-Gal) staining assay and cell proliferation. Treatment with nanomolar concentrations of DOX (5–100 nM) for 3 h resulted in a dose-dependent increase in cellular senescence by 72 h, as evidenced by a significant increase in the proportion of SA-β-Gal staining (Figure 1B,C). Specifically, 100 nM DOX induced SA-β-Gal expression in 77 ± 10% of the population, while 50 nM DOX resulted in 50 ± 1.8% of SA-β-Gal positive cells (Figure 1C). In parallel, DOX treatment led to a progressive decline in cell growth across the same concentration range, with the reduction in cell number reaching a near-maximal effect at 50 nM (Figure 1D). Notably, Annexin V and PI dual staining and flow cytometry assay showed that 50 nM DOX did not induce apoptosis (Figure S1). Taken together, these data indicate that nanomolar concentrations of DOX effectively trigger H9C2 cell senescence, with 50 nM eliciting a half-maximal effect on SA-β-Gal positive and a maximal effect on growth retardation. Consequently, the 50 nM DOX concentration was selected for all subsequent experiments to characterize the senescent phenotype and evaluate therapeutic interventions.

### 3.2. DOX Induces a Comprehensive Suite of Senescence Markers in H9C2 Cells

**Figure 2:**
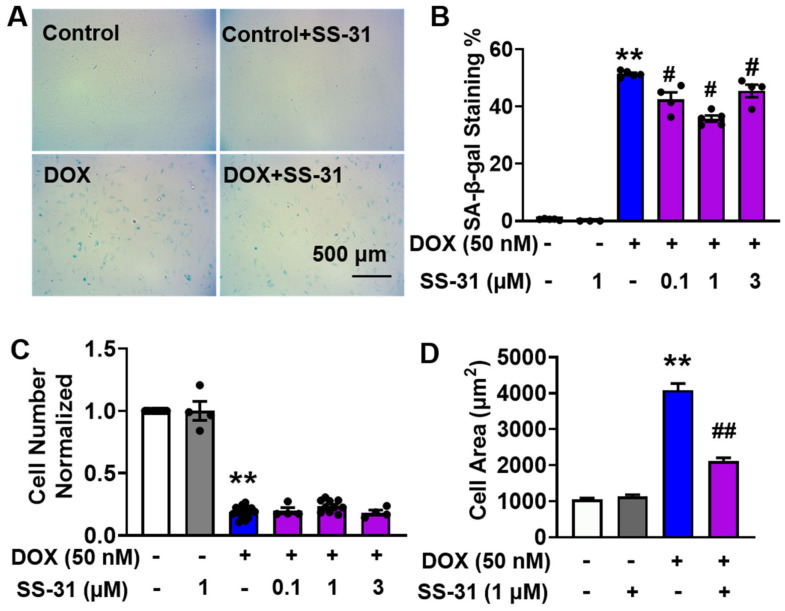
SS-31 partially mitigates the DOX-induced senescence in H9C2 cells. (**A**) Representative images of SA-β-gal staining in DOX-exposed H9C2 cells with or without SS-31 treatment. Scale bar: 500 μm. (**B**) Quantification of SA-β-gal positive cells following treatment with 50 nM DOX and varying concentrations of SS-31 (0.1, 1, and 3 µM), N = 3–5. (**C**) Effects of SS-31 on DOX-induced cell growth inhibition. SS-31 treatment did not significantly reverse the DOX-induced growth inhibition, N = 4–14. (**D**) Quantification of cell area. 50 nM DOX treatment significantly induced cell hypertrophy, which was markedly attenuated by SS-31. N = 42–59 cells from three independent experiments. In (**B**,**C**), each black dot represents a single independent experiment. Open box, control; Grey box, SS-31 only; Blue box, DOX only; Purple box, DOX plus SS-31 at the concentration indicated. All the data are mean ± SEM; ** *p* < 0.01 vs. control, # *p* < 0.05 vs. DOX, and ## *p* < 0.01 vs. DOX.

**Figure 3:**
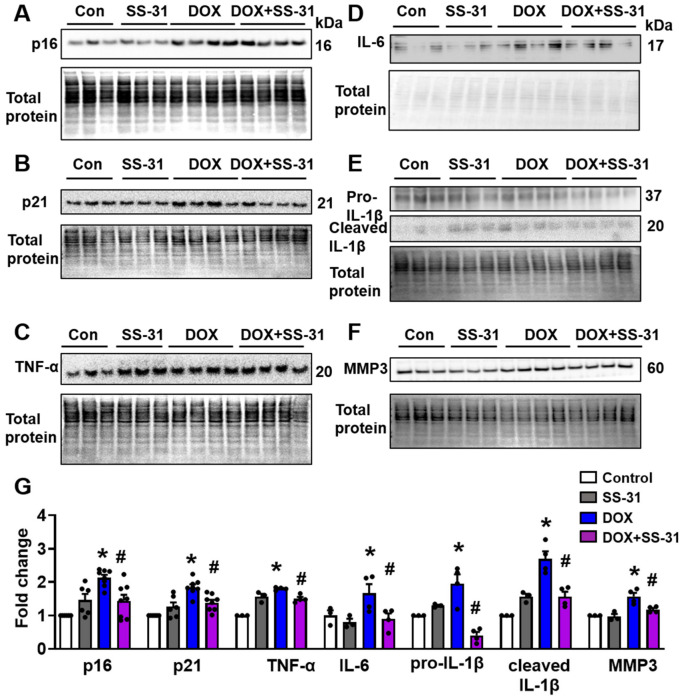
SS-31 attenuates the expression of molecular senescence markers and SASP components in DOX-treated H9C2 cells. H9C2 cells were exposed to 50 nM DOX for 3 h, followed by treatment with 1 µM SS-31 for 48 or 72 h. Representative Western blot images and corresponding quantifications are shown for: (**A**) p16, (**B**) p21, (**C**) TNF-α, (**D**) IL-6, (**E**) IL-1β, and (**F**) MMP3. (**G**) Summary quantification of protein expression levels normalized to total protein. In (**G**), each black dot represents a single independent experiment. Open box, control; Grey box, SS-31 only; Blue box, DOX only; Purple box, DOX plus SS-31. N = 3–8. All the data are mean ± SEM, * *p* < 0.05 vs. control, # *p* < 0.05 vs. DOX.

**Figure 4:**
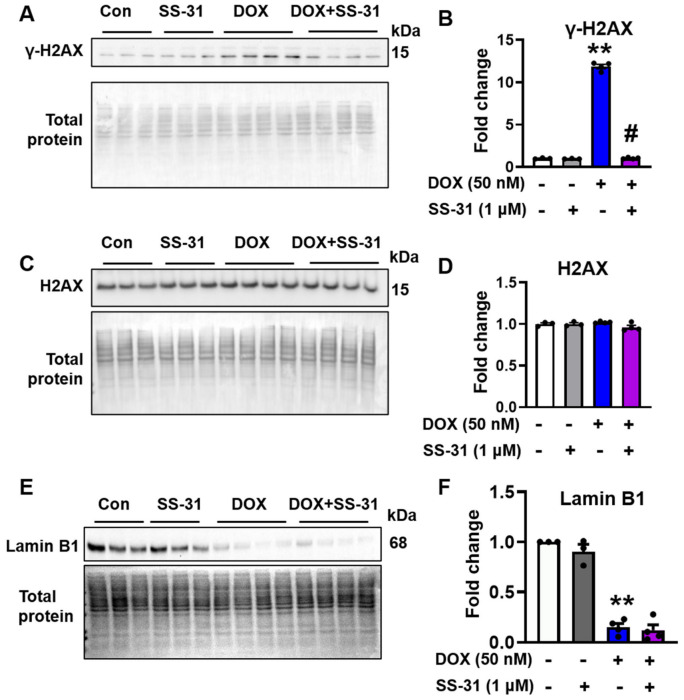
SS-31 reduces DOX-induced upregulation of γ-H2AX but does not rescue the DOX-induced downregulation of loss of Lamin B1. (**A**,**C**,**E**) Western blot image showing the expression levels of γ-H2AX, H2AX and Lamin B1 in H9C2 cells following treatment with 50 nM DOX in the presence or absence of 1 μM SS-31. (**B**,**D**,**F**) Quantification of γ-H2AX, H2AX, and Lamin B1 protein levels. Each black dot represents a single independent experiment. Open box, control; Grey box, SS-31 only; Blue box, DOX only; Purple box, DOX plus SS-31. N = 3–4 in each group. All the data are mean ± SEM, ** *p* < 0.01 vs. control, # *p* < 0.05 vs. DOX.

Beyond SA-β-Gal activity staining (Figure 1B,C and Figure 2A,B) and growth inhibition (Figure 1D and Figure 2C), a 3 h treatment with 50 nM DOX elicited several additional hallmarks of cellular senescence. By day 3 post-treatment, DOX-treated H9C2 cells exhibited an increased cell area, consistent with the flattened/enlarged morphology of senescent cells (Figure 2D) [34]. Furthermore, Western blot analysis revealed that 50 nM DOX significantly upregulated the cyclin-dependent kinase inhibitors p16 and p21 (Figure 3A,B,G. All original scans of the Western blot images are in a PPTX file as Supplementary Materials). We also observed an induction of the senescence-associated secretory phenotype (SASP), characterized by increased expression of pro-inflammatory cytokines TNFα, IL-6, IL-1β (both pro- and cleaved form) (Figure 3C–E,G), as well as the matrix metalloproteinases 3 (MMP3) (Figure 3F,G). Moreover, 50 nM DOX increased γH2AX (Figure 4A,B), a key marker of cellular senescence, suggesting persistent DNA double-strand breaks and the activation of the DNA damage response. The total H2AX was not changed with 50 nM DOX treatment (Figure 4C,D). Additionally, we observed a downregulation of Lamin B1 (Figure 4E,F), a marker of nuclear envelope integrity that typically declines during senescence.

**Figure 5:**
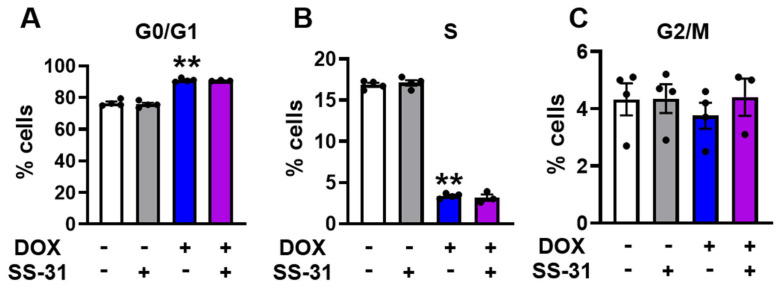
SS-31 does not reverse the cell cycle arrest induced by 50 nM DOX. Quantitative analysis of the percentage of cells in the (**A**) G0/G1 phase, (**B**) S phase, and (**C**) G2/M phase. Each black dot represents a single independent experiment. Open box, control; Grey box, SS-31 only; Blue box, DOX only; Purple box, DOX plus SS-31. N = 4. All the data are mean ± SEM, ** *p* < 0.01 vs. control.

For a dividing cell, a defining feature of cellular senescence is irreversible cell cycle arrest. While previous studies have reported S-phase [13] or G2 phase [35] arrest following 100 nM DOX exposure, our flow cytometric analysis using EdU pause incorporation and PI staining demonstrated that 50 nM DOX significantly increased the fraction of cells in G0/G1 phase from 76.5 ± 0.0% to 91.0 ± 0.0% (Figure 5A) but decreased the fractions of cells in the S phase from 16.9 ± 0.0% to 3.4 ± 0.0% (Figure 5B), reflecting impaired cell cycle progression. Correspondingly, the G2/M phase remained the same in all the groups (Figure 5C).

**Figure 6:**
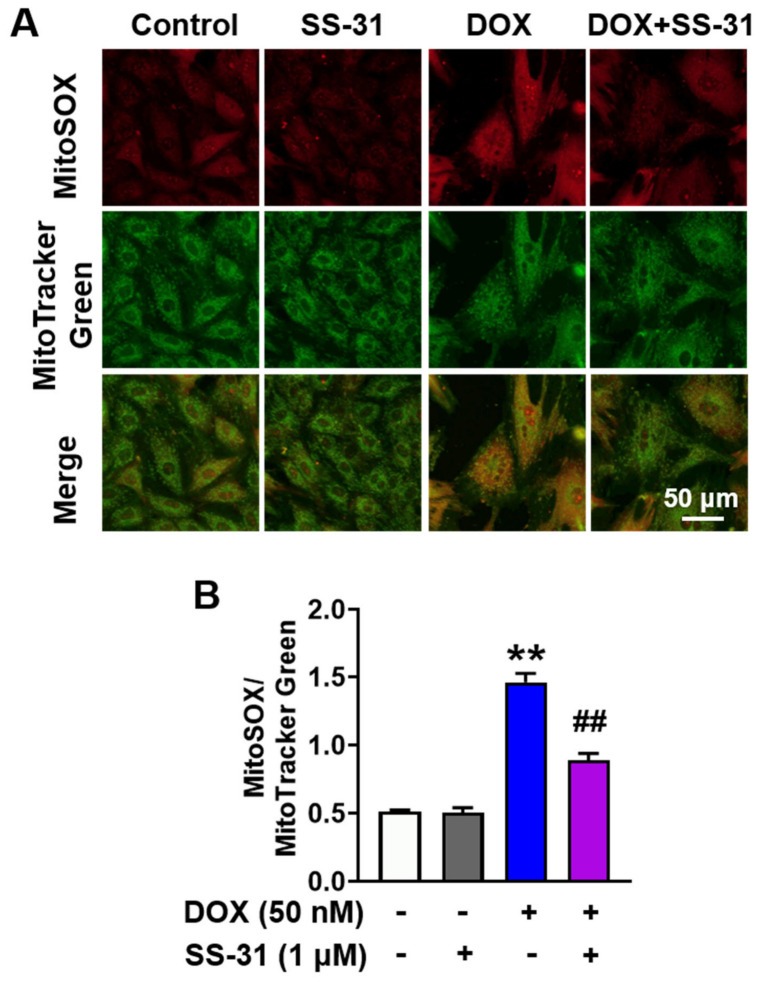
SS-31 mitigates DOX-induced mitochondrial superoxide production. (**A**) Representative confocal microscopy images of H9C2 cells stained for mitochondrial superoxide. Upper panel, MitoSOX; middle panels, MitoTracker Green; lower panel, merge of MitoSOX and MitoTracker Green. Cells were treated with 50 nM DOX in the presence or absence of 1 μM SS-31. Scale bar: 50 μm. (**B**) Quantification of mitochondrial superoxide levels, expressed as the fluorescence intensity ratio of MitoSOX to MitoTracker Green. Open box, control; Grey box, SS-31 only; Blue box, DOX only; Purple box, DOX plus SS-31. N = 36–55 cells from three independent preparations. All the data are mean ± SEM, ** *p* < 0.01 vs. control, ## *p* < 0.01 vs. DOX.

Mitochondrial dysfunction, specifically increased reactive oxygen species (ROS) production, is another established hallmark of senescence [34]. Using MitoSOX normalized to MitoTrackerGreen to account for mitochondrial mass, we found that 50 nM DOX significantly elevated mitochondrial superoxide levels (Figure 6A,B). Collectively, these data provide a detailed characterization of a 50 nM DOX-induced H9C2 cell senescence model, establishing a robust platform for mechanistic studies and drug screening.

### 3.3. SS-31 Attenuates 50 nM DOX-Induced H9C2 Cell Senescence Without Reversing Cell Cycle Arrest

The unlimited expansion capacity of the H9C2 cell makes this low-concentration DOX-induced senescence model a feasible platform for screening cardioprotective compounds. In the present study, we evaluated whether the mitochondrial-targeted tetrapeptide SS-31 (elamipretide) could mitigate the senescent phenotype. H9C2 cells exposed to 50 nM DOX were treated with varying concentrations of SS-31 (0.1, 1, and 3 μM) according to the protocol in Figure 1A. SS-31 significantly reduced the proportion of SA-β-gal-positive cells at all three concentrations (Figure 2A,B), with a maximum reduction observed at 1 μM, decreasing from 51.4 ± 0.5% to 35.8 ± 1.0% (Figure 2B). Moreover, 1 μM SS-31 had a maximal effect, and 3 μM and 10 μM of SS-31 had no further protection, on decreasing the senescence markers p16 and p21 (Figure S2, the original scans of the Western blot images are in a PPTX file as Supplementary Materials), and mitochondrial ROS (Figure S3). Notably, none of the tested concentrations reversed the cell growth retardation induced by DOX (Figure 2C). Based on these results, 1 μM SS-31 treatment was selected for further mechanistic evaluation.

At 1 μM, SS-31 effectively attenuated the morphological and molecular hallmarks of senescence. It significantly reduced the DOX-induced cell hypertrophy, decreasing the cell area by approximately 50% (from 4086 ± 164 µm^2^ to 2116 ± 89 µm^2^, Figure 2D). Furthermore, 1 μM SS-31 treatment downregulated the protein expression of the canonical senescence markers p16 and p21 (Figure 3A,B,G), and suppressed the induction of SASP markers, including TNF-α, IL-6, IL-1β (pro- and cleaved forms), and MMP3 (Figure 3C–G). 1 μM SS-31 also decreased γ-H2AX, another senescence marker (Figure 4A,B).

Because SS-31 is known to incorporate into cardiolipin in the inner mitochondrial membrane to enhance the electron transport efficiency and reduce the ROS production [36], we examined its effect on DOX-induced ROS production. Indeed, 1 μM SS-31 significantly decreased the MitoSOX/MitoTracker Green ratio, indicating a reduction in mitochondrial superoxide production (Figure 6A,B).

Unexpectedly, despite its effects on the morphology and protein markers, 1 μM SS-31 treatment failed to alleviate the cell cycle arrest induced by 50 nM DOX (Figure 5). Additionally, SS-31 did not prevent the 50 nM DOX-induced downregulation of Lamin B1 (Figure 4E,F), which aligns with its inability to rescue cell growth retardation (Figure 2C). Taken together, these findings demonstrated that while SS-31 attenuates mitochondrial ROS and SASP features of 50 nM DOX-induced senescence, it does not bypass the cell cycle arrest.

### 3.4. SS-31 Attenuates the DOX-Induced Elevation of Mitochondrial Respiration

**Figure 7:**
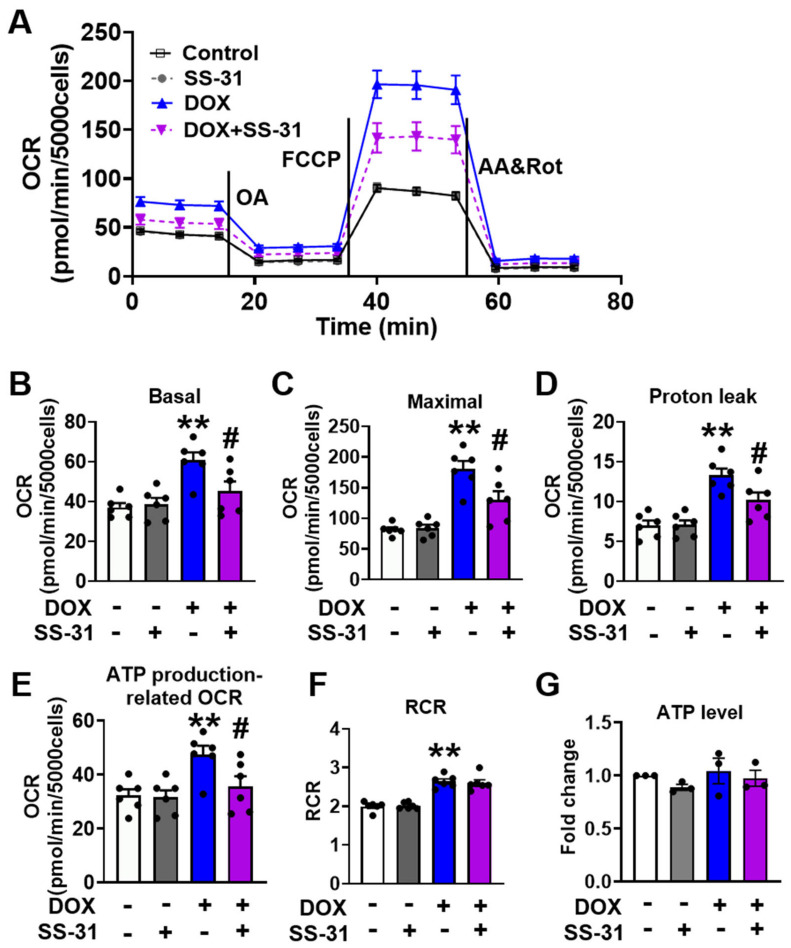
SS-31 attenuates the DOX-induced elevation of mitochondrial respiration. (**A**) Average traces of oxygen consumption rate (OCR) in intact H9C2 cells using Seahorse XF96 mitochondrial stress assay. The vertical lines indicate the sequential injection of oligomycin A, (OA, 5 µM), carbonyl cyanide-p-trifluoromethoxyphenylhydrazone (FCCP, 10 µM), and a combination of antimycin A (AA, 5 µM) and rotenone (Rot, 5 µM). (**B**–**F**) Quantification of mitochondrial respiration parameters, including (**B**) basal respiration, (**C**) maximal respiration, (**D**) proton leak-related respiration, (**E**) ATP production-related respiration, and (**F**) respiration control ratio (RCR), calculated as the ratio of maximal to basal OCR. N = 6 independent experiments. (**G**) Quantification of total cellular ATP levels. Data were normalized to control cells to represent relative changes in ATP content. N = 13 measurements from 3 independent preparations. Each black dot represents a single independent experiment. Open box, control; Grey box, SS-31 only; Blue box, DOX only; Purple box, DOX plus SS-31. All the data are mean ± SEM, ** *p* < 0.01 vs. control, # *p* < 0.05 vs. DOX.

**Figure 8:**
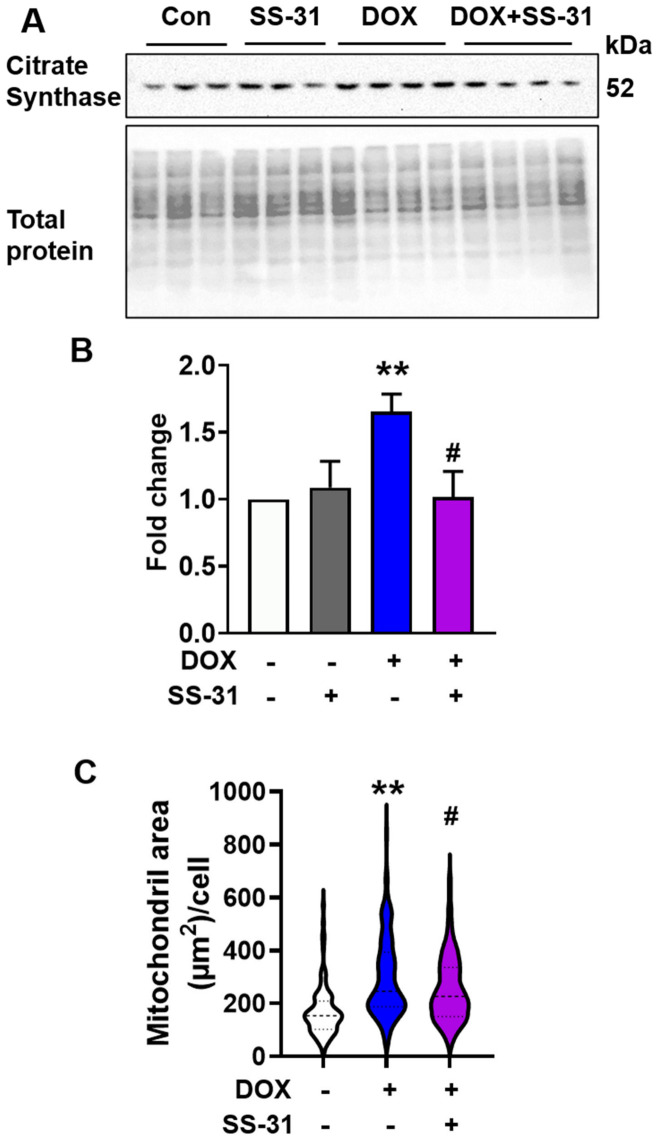
SS-31 suppresses the DOX-induced increase in citrate synthase expression and mitochondrial area. (**A**) Western blot image of citrate synthase in H9C2 cells following exposure to 50 nM DOX with or without 1 μM SS-31. (**B**) Quantification of citrate synthase protein levels. N = 3–4. All the data are mean ± SEM. (**C**) SS-31 suppresses the DOX-induced increase in mitochondrial area. N = 103–107 cells from 5 independent preparations. The DOX-induced elevation of citrate synthase and mitochondrial area, indicative of increased mitochondrial mass, was significantly attenuated by SS-31 treatment. Open box, control; Grey box, SS-31 only; Blue box, DOX only; Purple box, DOX plus SS-31. ** *p* < 0.01 vs. control, # *p* < 0.05 vs. DOX.

We next investigated whether 50 nM DOX impairs mitochondrial respiration and whether SS-31 could mitigate these changes. In contrast to the previous report, where high-dose DOX (5 µM for 24 h) decreased basal and maximal mitochondrial respiration [32], we found that a 3 h treatment with 50 nM DOX significantly increased both basal and maximal oxygen consumption rates (OCR) (Figure 7A–C). Furthermore, DOX augmented proton leak-related mitochondrial respiration (Figure 7D), a phenomenon similarly observed in aged cardiomyocytes [29]. The 50 nM DOX increased ATP production-related respiration (Figure 7E), which may be driven by an increase in mitochondrial mass, as evidenced by upregulated citrate synthase levels (Figure 8A,B) and mitochondrial area (Figure 8C), similar to the previous report [37]. However, the total ATP level (Figure 7G) and mitochondrial membrane potential (Figure S4) remained unchanged in the DOX treatment cells compared to controls. This discrepancy between elevated ATP production-related respiration and static ATP levels suggests a reduction in mitochondrial oxidative phosphorylation efficiency. Intriguingly, 50 nM DOX also increased the mitochondrial respiration control ratio (RCR), calculated as the ratio of maximal OCR (induced by FCCP) to basal OCR (Figure 7F). Despite these functional shifts, no significant changes were detected in the protein expression levels of the five mitochondrial respiratory complexes (Figure S5, all original scans of the Western blot images are in a PPTX file as Supplementary Materials).

Treatment with 1 µM SS-31 significantly attenuated the DOX-induced elevations in basal (Figure 7B) and maximal respiration (Figure 7C). SS-31 also mitigated the excessive mitochondrial proton leak induced by 50 nM DOX (Figure 7D). To be noted that, while SS-31 decreased ATP production-related respiration (Figure 7E), it maintained ATP levels equivalent to those of the control and DOX only groups (Figure 7G). In summary, 50 nM DOX induces a hyper-metabolic but inefficient respiratory pattern, which SS-31 effectively shifts back toward baseline homeostatic levels.

## 4. Discussion

### 4.1. A Robust Model of DOX-Induced Cardiomyocyte Senescence

Previous studies have established that sub-lethal concentrations of DOX, typically around 100 nM, induce senescence in H9C2 cells following exposure durations ranging from 48 h [11,38], 24 h [39], or as briefly as 3 h followed by 48 h culture [13,14,40]. In this study, we demonstrated that a short-term 3 h exposure to even lower concentrations of DOX triggers senescence in a dose-dependent manner. Specifically, we provided multiple lines of evidence confirming that 50 nM DOX effectively induces a senescence phenotype in H9C2 cells. First, consistent with the earlier report [11], 50 nM DOX resulted in a high percentage of positive SA-β-gal staining. Second, this treatment led to cell growth retardation, a hallmark of senescence in an immortalized cell line [34]. Third, the cell exhibited significant hypertrophy. Fourth, the upregulation of the molecular senescence markers p16 and p21, alongside a robust SASP marker, as well as γH2AX. Fifth, DOX treatment increased the senescence marker ROS production [34]. Sixth, it led to the loss of nuclear Lamin B1 level. Finally, 50 nM DOX increased mitochondrial proton leak, a functional deficit previously reported in intact aged cardiomyocytes [29] and in mitochondria isolated from aged cardiac tissue [41]. This model is particularly valuable for investigating the mechanisms underlying the delayed or late-stage cardiomyocyte dysfunction often observed in pediatric cancer survivors [42]. Furthermore, given that H9C2 cells are immortal cells, this model provides a robust, scalable, and feasible platform for high-throughput screening of therapeutic compounds aimed at mitigating DOX-induced cardiomyocyte senescence. The similar low-concentration DOX-induced cardiomyocyte senescence is reported in neonatal cardiomyocytes by 100 nM [13] and 50 nM for 3 days [15], and in hiPSC differentiated cardiomyocytes by 50 nM for 3 days [16].

### 4.2. Mechanistic Insights into SS-31-Mediated Protection

The major finding of this study is that SS-31 effectively mitigates DOX-induced senescence phenotypes without reversing the associated cell cycle arrest or growth inhibition. Consistent with previous reports [39], we demonstrated that DOX triggers excessive ROS production in H9C2 cells. This oxidative stress is a known driver of DNA and macromolecular damage [43], signaling transduction dysregulation, Ca^2+^ mishandling [44], and overall cellular dysfunction, ultimately accelerating senescence transition [45]. Our results indicate that SS-31 prevents DOX-induced mitochondrial ROS overproduction, which likely serves as a primary mechanism for its protective effects. SS-31 decreases oxidative stress, which might change the molecular modification of the Ca^2+^ channels and pumps, and thus improves the Ca^2+^ handling, including Ca^2+^ spark and Ca^2+^ transient [44]. Furthermore, while SS-31 decreases ATP production-related respiration in DOX-treated cells, intracellular ATP levels remained stable. This finding suggests that SS-31 improves mitochondrial oxidative phosphorylation efficiency, allowing the cell to maintain energy homeostasis with lower oxygen consumption.

Mechanistically, SS-31 is preferentially incorporated into cardiolipin within the mitochondrial inner membrane. This interaction stabilizes cristae curvature and respiratory complexes, may prevent cytochrome C release, and decreases the electron leak and superoxide production [22]. The reduction in ROS production could change the gene expression profile, protein modification, signaling pathway and cell metabolism, which need further investigation by RNA sequencing, proteomics and metabolomics.

From a translational perspective, the finding that SS-31 does not reverse DOX-induced growth retardation or cell cycle arrest is significant. This suggests that SS-31 might not interfere with the anti-neoplastic efficacy of DOX, while simultaneously protecting cardiomyocytes from the detrimental effects of cellular senescence and damage; however, this needs further verification on the cancer cell or *in vivo* animal model carrying a tumor.

### 4.3. Metabolic Compensation and Mitochondrial Inefficiency

In contrast to the respiratory depression previously observed with high concentrations (5 µM) of DOX [32], our findings reveal that a low concentration of DOX (50 nM) significantly increases basal and maximal mitochondrial respiration in the H9C2 cells. This hyper-metabolic state aligns with reports of increased respiration in A549 lung cancer cells following exposure to 100 nM DOX for 48 h [35].

Notably, the DOX-induced increased mitochondrial proton leak observed in our study recapitulates the excessive mitochondrial proton leak in aged cardiomyocytes [29] and mitochondria [41]. An excessive mitochondrial proton leak imposes a significant burden on mitochondrial workload, reducing respiratory efficiency and promoting electron leakage, which in turn exacerbates superoxide generation [46]. To compensate for this inefficiency and maintain the mitochondrial membrane potential to generate ATP, H9C2 cells appear to upregulate the mitochondrial respiratory rate, as reflected by the increased RCR observed after 50 nM DOX exposure [33,47]. While we detected an increase in citrate synthase protein levels, the abundance of mitochondrial respiratory complexes remained unchanged. This suggests that the induction of mitochondrial respiration in this model may be driven by mitochondrial mass or qualitative function shifts rather than an increase in the density of respiratory chain components, as previous reported [48,49]. The precise mechanisms underlying this respiratory induction, particularly the elevation of the RCR, warrant further investigation.

### 4.4. Study Limitations and Future Directions

Several limitations of the present study should be acknowledged. First, our findings are based on an in vitro model of DOX-induced senescence in H9C2 cardiomyocytes. While this provides a controlled environment for mechanistic study, it will be essential to validate these observations *in vivo*. Specifically, future studies should evaluate the effects of chronic, low-dose DOX treatment on cardiac senescence in animal models, especially tumor-bearing models, to better simulate the clinical environment and confirm the translational potential of our findings.

Second, although we established that SS-31 reduces ROS production in this low-concentration DOX model, the exact mechanisms by which this reduction translates into anti-senescence protection remain to be fully elucidated. The prevention of the senescence phenotype likely involves complex downstream shifts, including altered post-translational modifications, the modulation of specific signaling pathways, and broader proteomic changes. In the signaling pathway perspective, the ROS/NLRP3 [50] and NF-κB pathways [51] may participate in the DOX-induced cardiotoxicity and SASP genes, including *IL-6*, *IL-1β*, and *MMP3*. The broad view of the mechanisms is the mitophagy [52], mitochondrial complexes activity [53], and the crosstalk between mitochondrial dysfunction and fatty acid metabolism [54]. Further investigation is required to map these downstream pathways and determine how mitochondrial stabilization by SS-31 ultimately intercepts the signaling cascades that drive cellular aging.

## 5. Conclusions

In summary, we have established and characterized a robust model of H9C2 cardiomyocyte senescence induced by a 3 h exposure to a sub-lethal (50 nM) concentration of DOX. This model effectively recapitulates the molecular, morphological, and functional hallmarks of cardiomyocyte aging, providing a feasible and scalable platform for mechanistic investigation and high-throughput drug screening. Furthermore, our findings demonstrate that mitochondrial-targeted tetrapeptide SS-31 attenuates the DOX-induced senescent phenotype and mitochondrial dysfunction without reversing the concomitant cell cycle arrest. These results offer novel insights into the pathways driving cardiac senescence and identify SS-31 as a promising therapeutic candidate for mitigating cardiotoxicity in patients undergoing DOX-based chemotherapy.

## Data Availability

The original contributions presented in the study are included in the article/Supplemental Materials; further inquiries can be directed to the corresponding authors.

## Supplementary Materials

The following supporting information can be downloaded at: https://www.mdpi.com/article/10.3390/biology15131034/s1, Figure S1. 50 nM DOX did not induce cell apoptosis. Figure S2. SS-31 dose effect on the DOX induced elevation of p16 and p21. Figure S3. SS-31 dose effect on the DOX induced elevation of mitochondrial superoxide production. Figure S4. 50 nM DOX did not decrease the mitochondrial membrane potential. Figure S5. 50 nM DOX does not alter the protein abundance of mitochondrial respiratory complexes. File S1. WB Images and condition final version.
