# Supplementary material for: Mitochondrial-Targeted SS-31 Attenuates the Doxorubicin-Induced Cardiomyoblast H9C2 Cell Senescence
Source: Biology (Basel). 2026 Jun 28;15(13):1034. doi: 10.3390/biology15131034 (PMC13359912; doi:10.3390/biology15131034)
Supplement: Supplementary file 1 [file biology-15-01034-s001.zip › biology-4322693 File S1-WB Images and condition final version .pptx]

## Slide 1
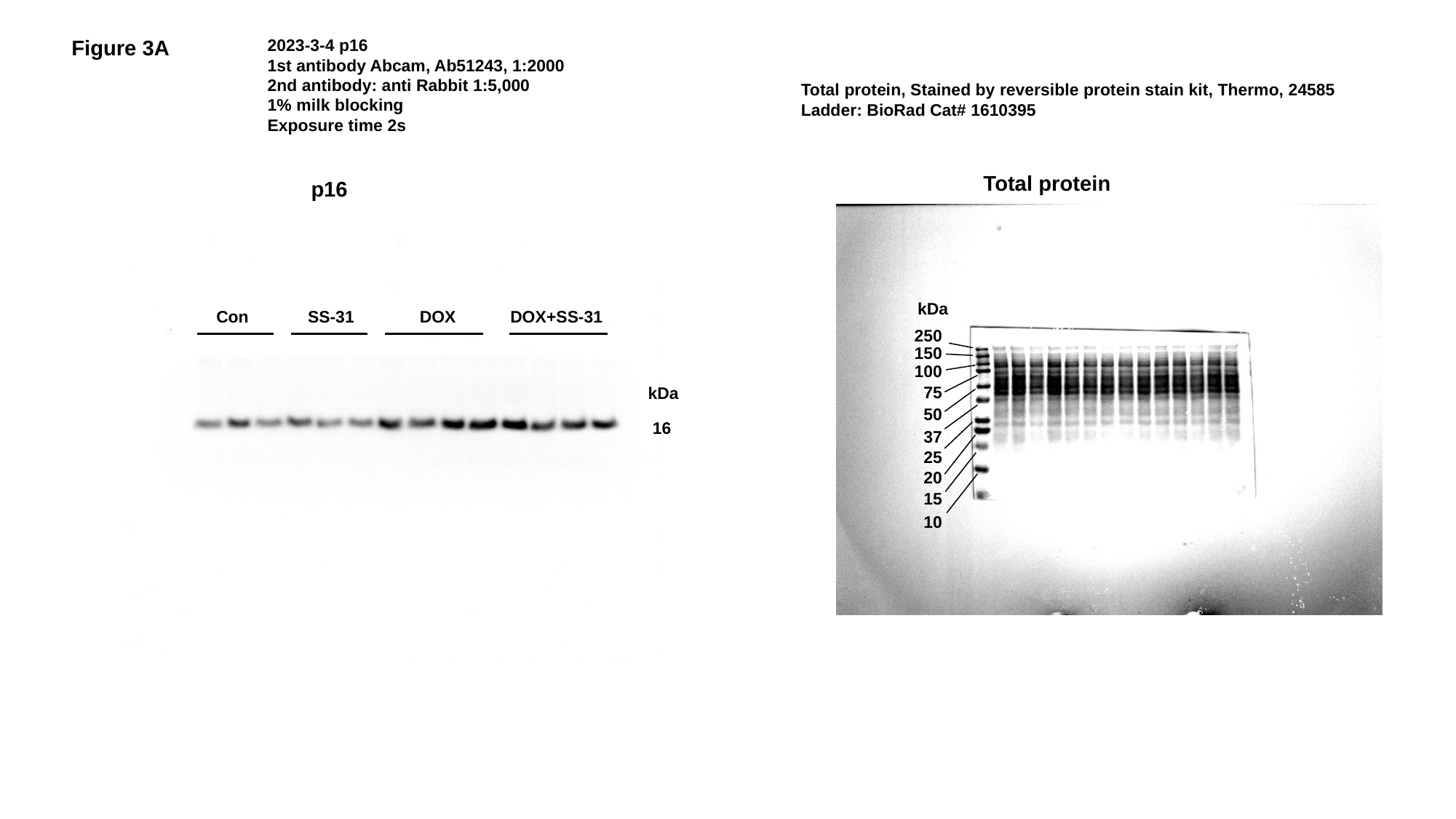

Figure 3A
2023-3-4 p16
1st antibody Abcam, Ab51243, 1:2000
2nd antibody: anti Rabbit 1:5,000
1% milk blocking
Exposure time 2s
Total protein, Stained by reversible protein stain kit, Thermo, 24585
Ladder: BioRad Cat# 1610395
Total protein
p16
kDa
Con
SS-31
DOX
DOX+SS-31
250
150
100
75
kDa
50
16
37
25
20
15
10

## Slide 2
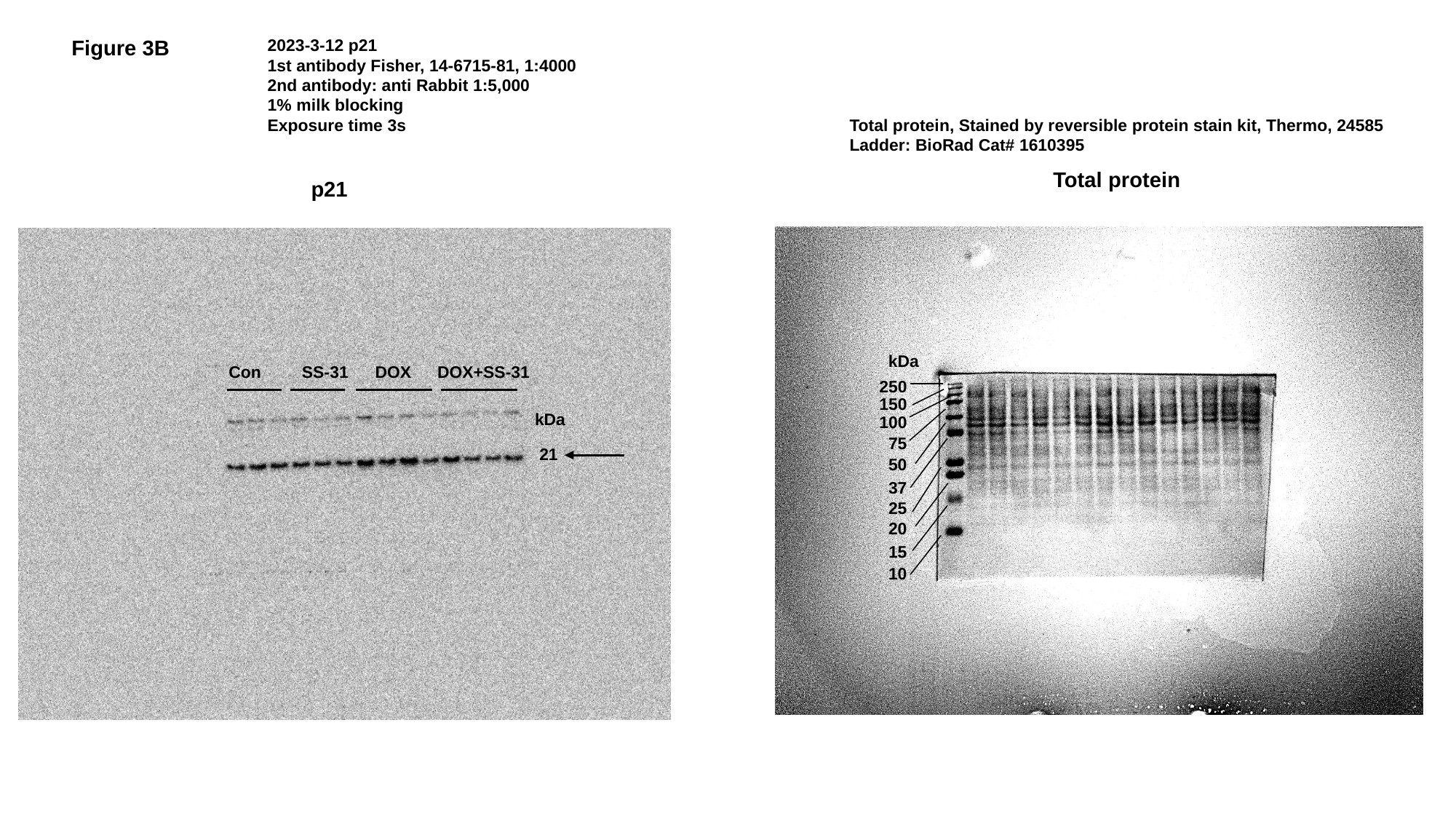

Figure 3B
2023-3-12 p21
1st antibody Fisher, 14-6715-81, 1:4000
2nd antibody: anti Rabbit 1:5,000
1% milk blocking
Exposure time 3s
Total protein, Stained by reversible protein stain kit, Thermo, 24585
Ladder: BioRad Cat# 1610395
Total protein
p21
kDa
Con
SS-31
DOX
DOX+SS-31
250
150
kDa
100
75
21
50
37
25
20
15
10

## Slide 3
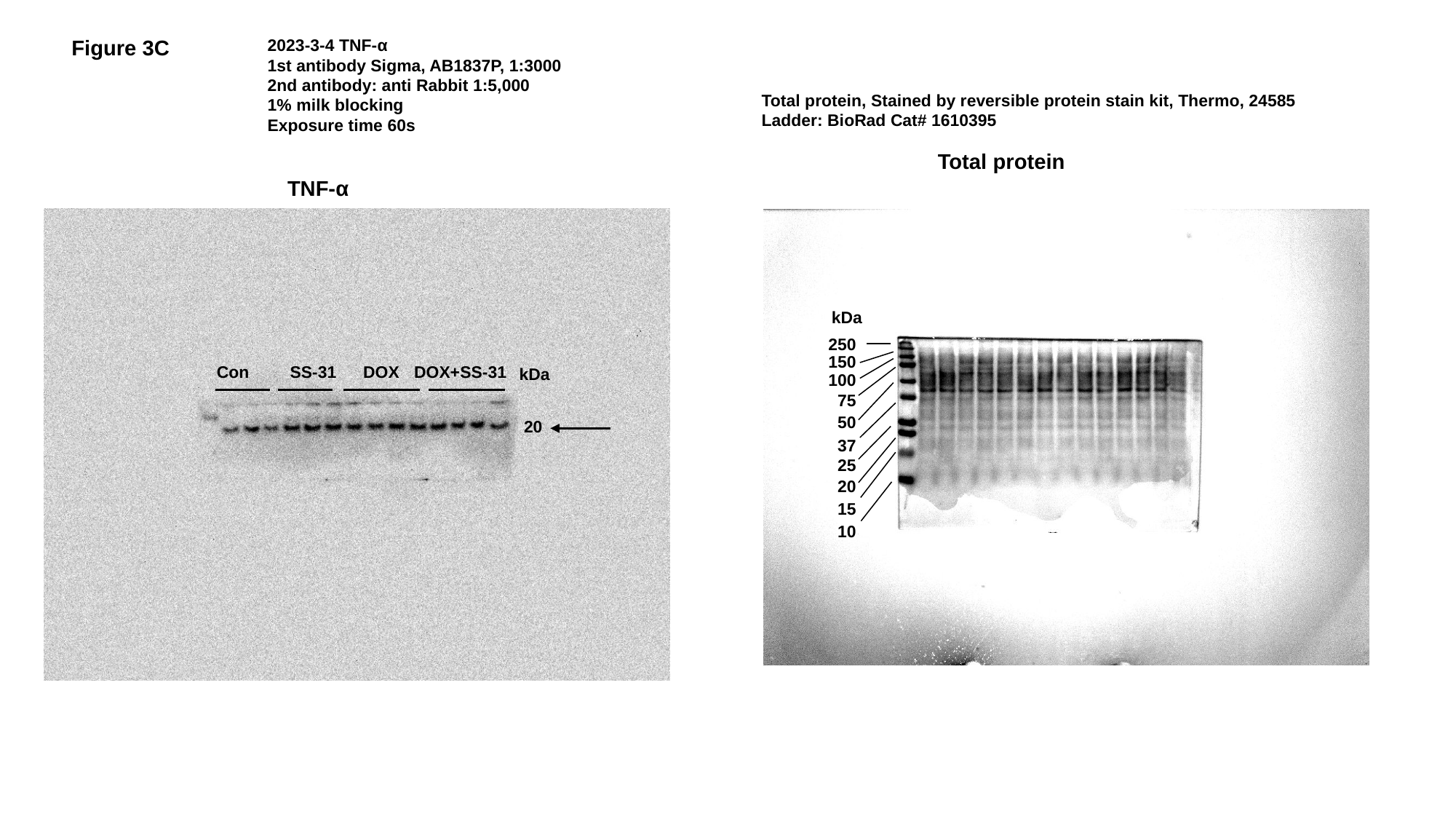

Figure 3C
2023-3-4 TNF-α
1st antibody Sigma, AB1837P, 1:3000
2nd antibody: anti Rabbit 1:5,000
1% milk blocking
Exposure time 60s
Total protein, Stained by reversible protein stain kit, Thermo, 24585
Ladder: BioRad Cat# 1610395
Total protein
TNF-α
kDa
250
150
kDa
Con
SS-31
DOX
DOX+SS-31
100
75
50
20
37
25
20
15
10

## Slide 4
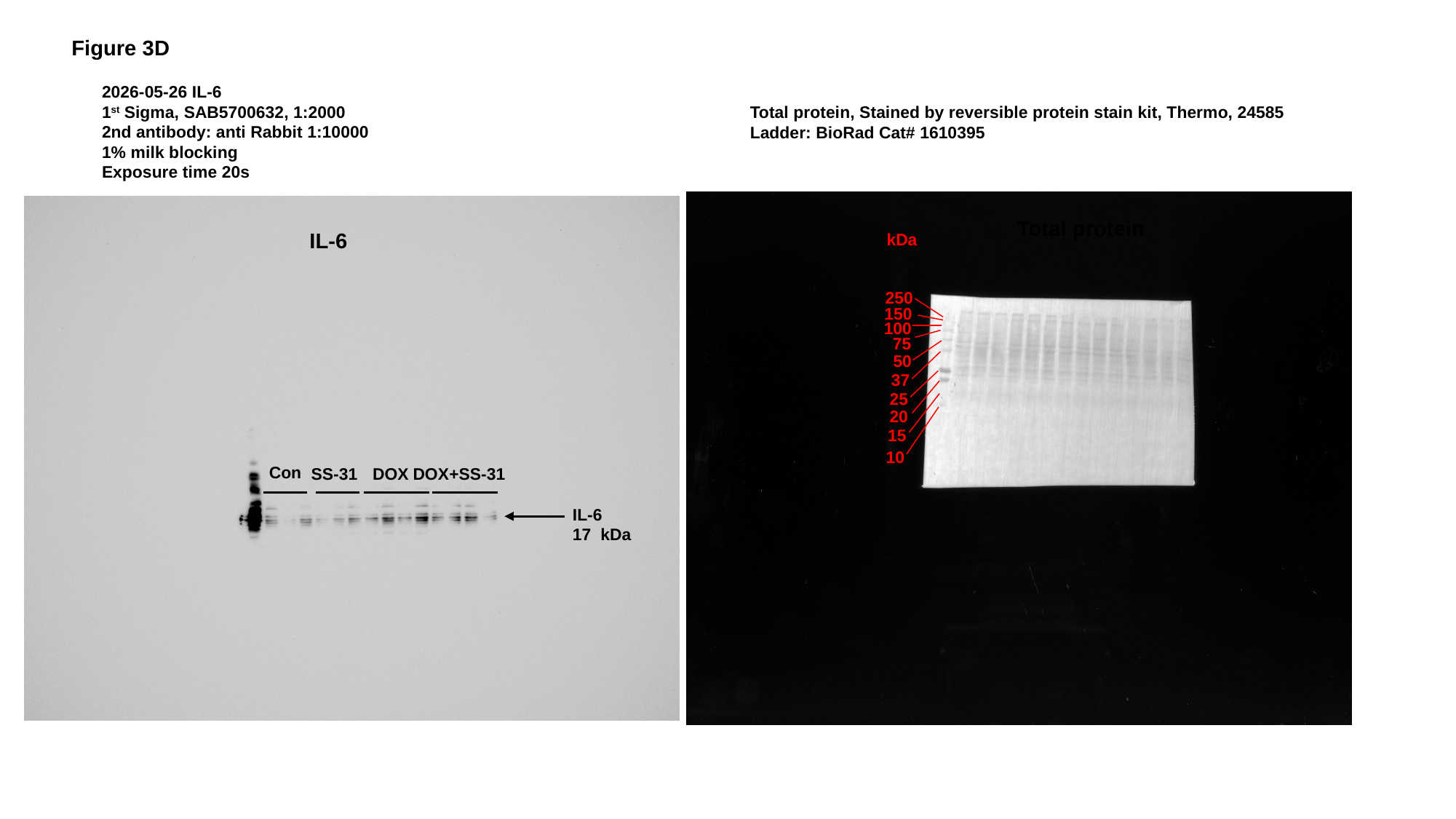

Figure 3D
2026-05-26 IL-6
1st Sigma, SAB5700632, 1:2000
2nd antibody: anti Rabbit 1:10000
1% milk blocking
Exposure time 20s
Total protein, Stained by reversible protein stain kit, Thermo, 24585
Ladder: BioRad Cat# 1610395
Total protein
kDa
IL-6
250
150
100
75
50
37
25
20
15
10
Con
SS-31
DOX
DOX+SS-31
IL-6
17 kDa

## Slide 5
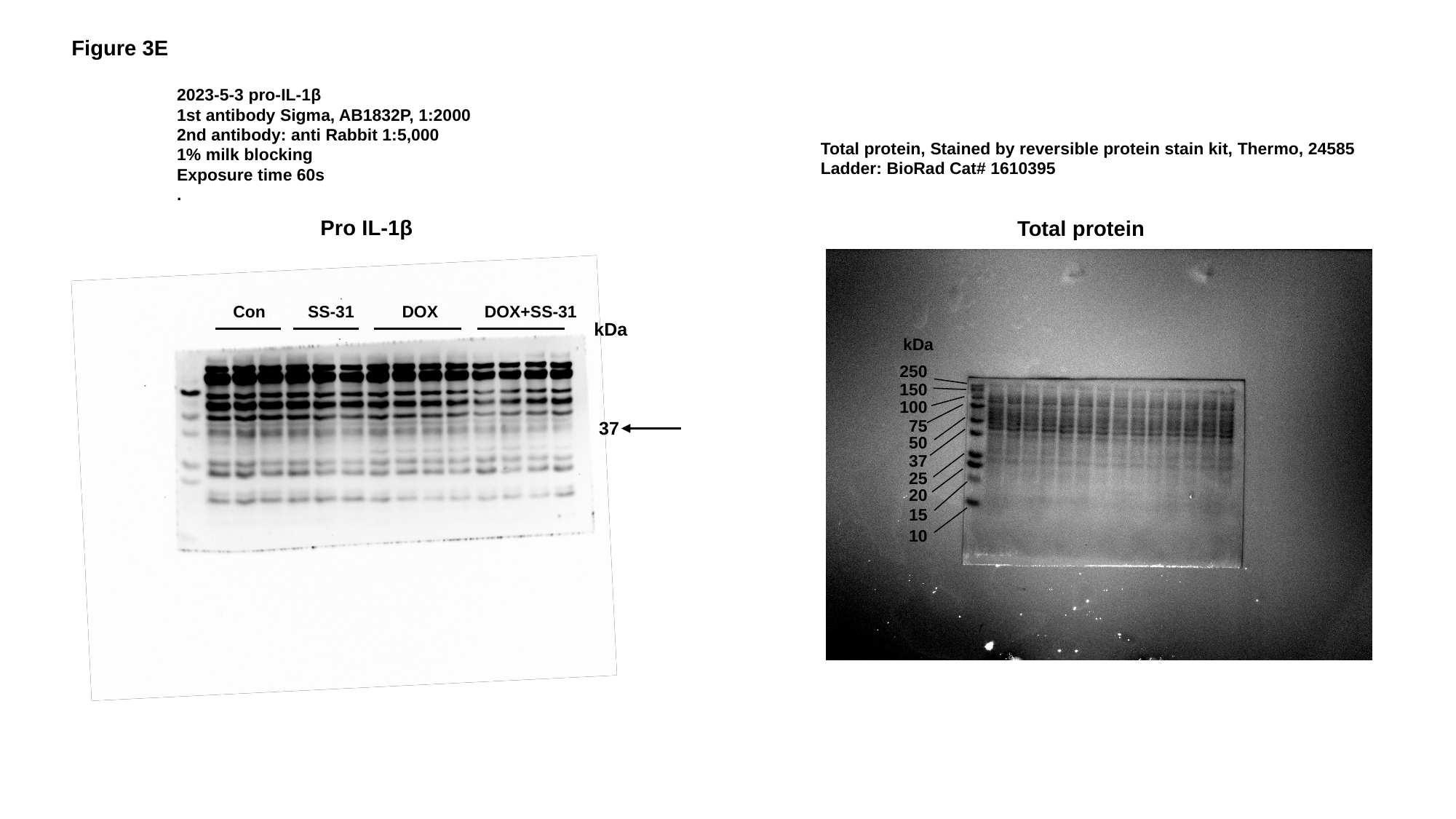

Figure 3E
2023-5-3 pro-IL-1β
1st antibody Sigma, AB1832P, 1:2000
2nd antibody: anti Rabbit 1:5,000
1% milk blocking
Exposure time 60s
.
Total protein, Stained by reversible protein stain kit, Thermo, 24585
Ladder: BioRad Cat# 1610395
Pro IL-1β
Total protein
Con
SS-31
DOX
DOX+SS-31
kDa
kDa
250
150
100
75
37
50
37
25
20
15
10

## Slide 6
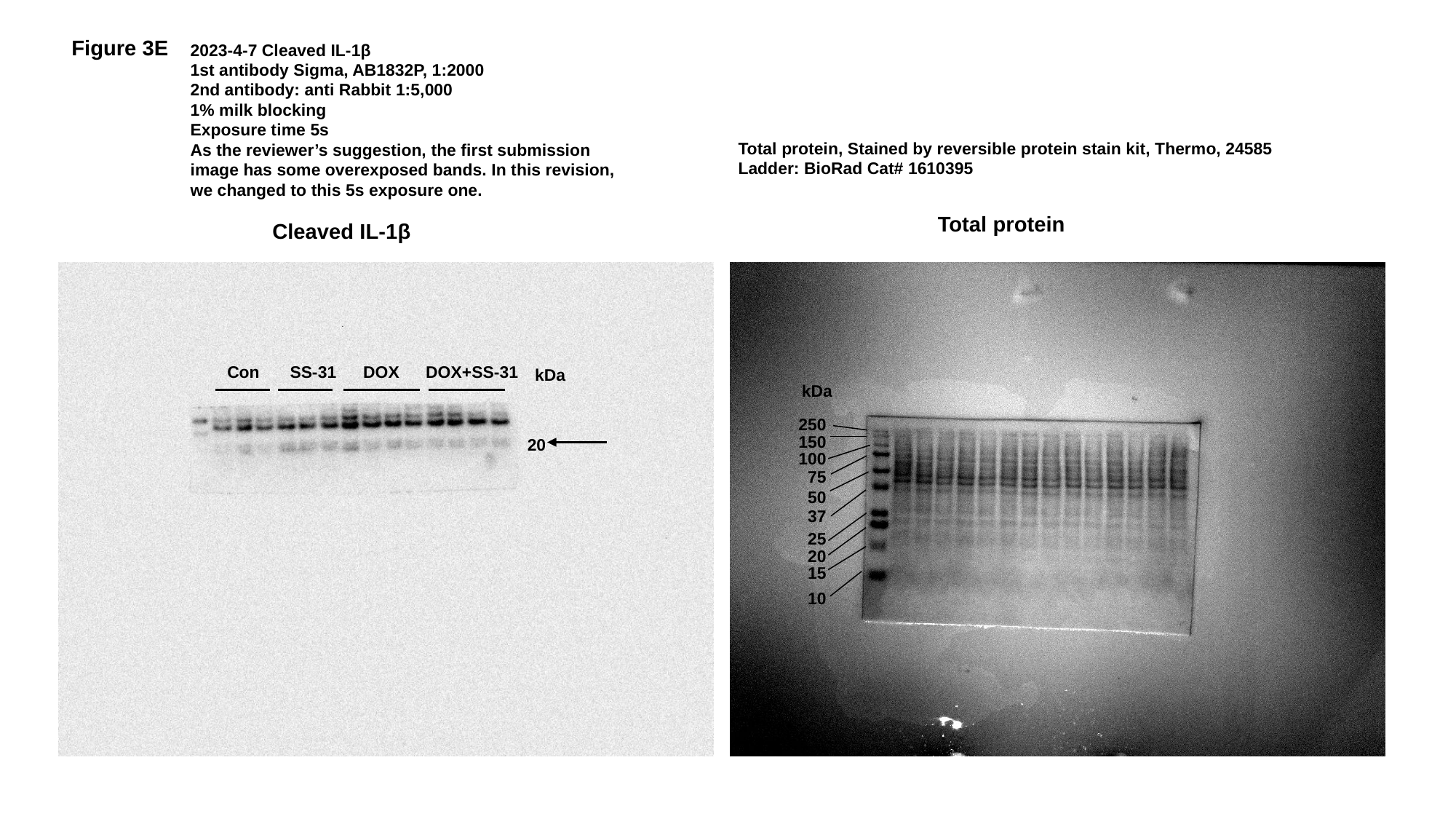

Figure 3E
2023-4-7 Cleaved IL-1β
1st antibody Sigma, AB1832P, 1:2000
2nd antibody: anti Rabbit 1:5,000
1% milk blocking
Exposure time 5s
As the reviewer’s suggestion, the first submission image has some overexposed bands. In this revision, we changed to this 5s exposure one.
Total protein, Stained by reversible protein stain kit, Thermo, 24585
Ladder: BioRad Cat# 1610395
Total protein
Cleaved IL-1β
kDa
Con
SS-31
DOX
DOX+SS-31
kDa
250
150
20
100
75
50
37
25
20
15
10

## Slide 7
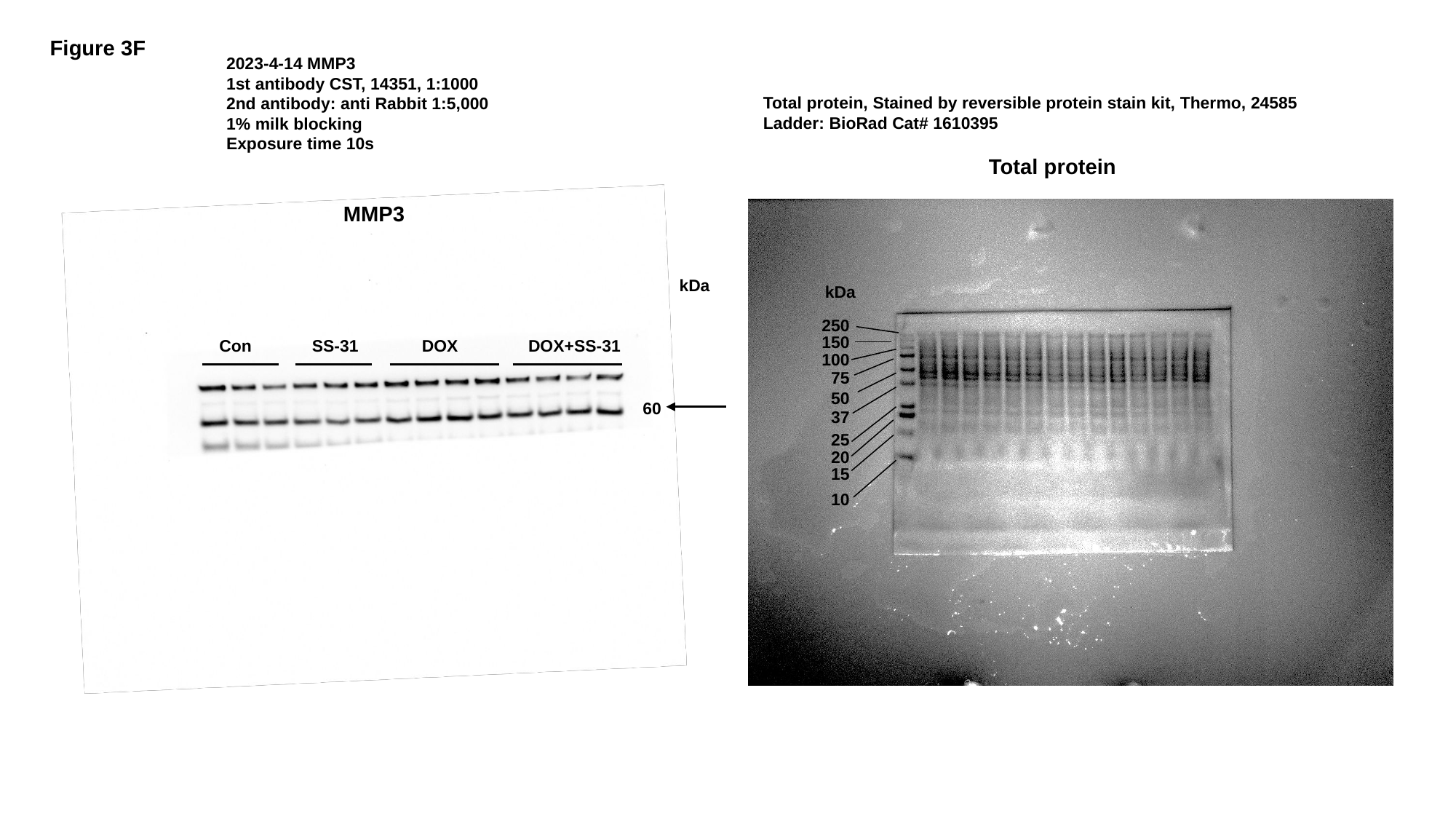

Figure 3F
2023-4-14 MMP3
1st antibody CST, 14351, 1:1000
2nd antibody: anti Rabbit 1:5,000
1% milk blocking
Exposure time 10s
Total protein, Stained by reversible protein stain kit, Thermo, 24585
Ladder: BioRad Cat# 1610395
Total protein
MMP3
kDa
kDa
250
150
Con
SS-31
DOX
DOX+SS-31
100
75
50
60
37
25
20
15
10

## Slide 8
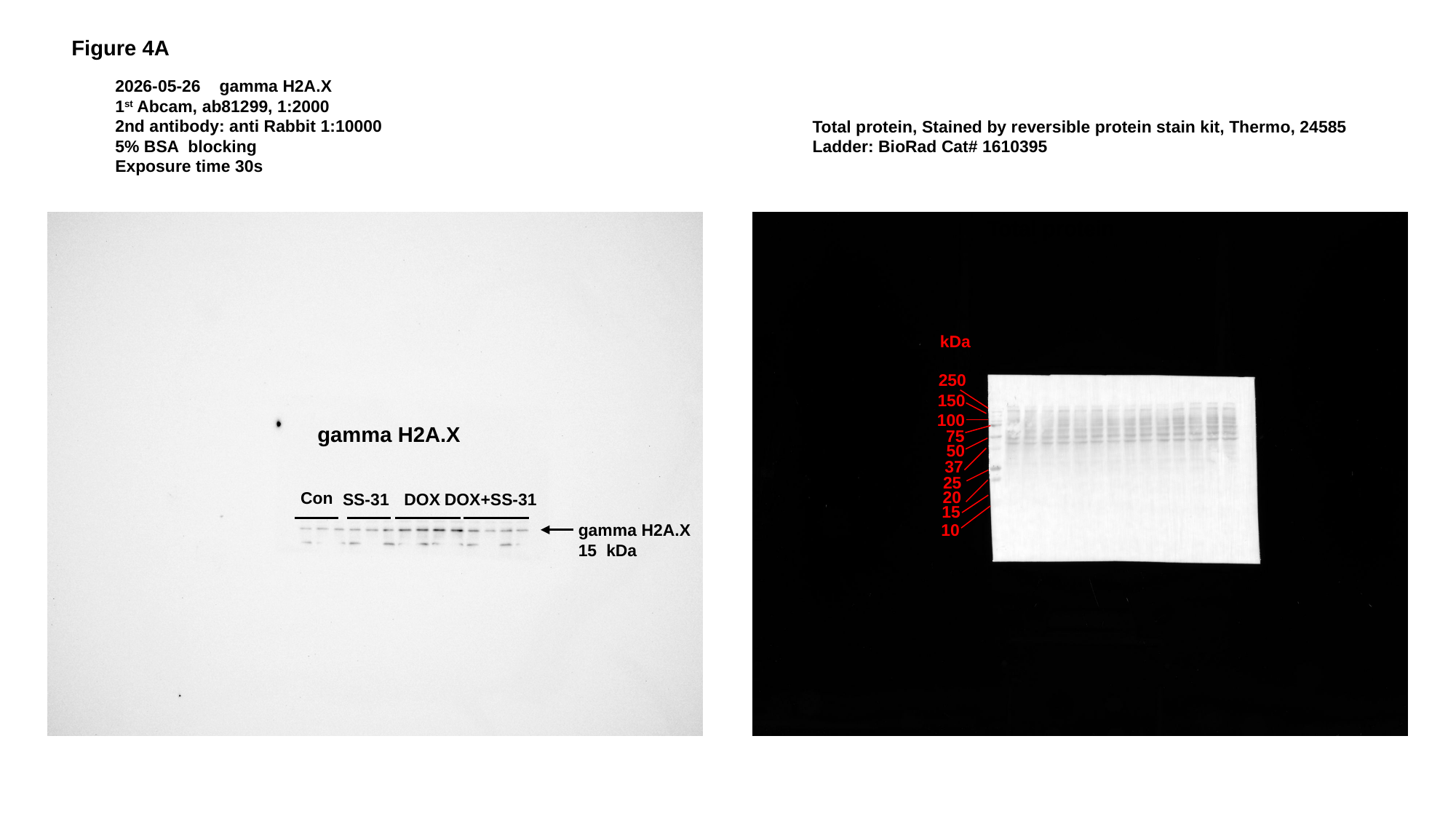

Figure 4A
2026-05-26 gamma H2A.X
1st Abcam, ab81299, 1:2000
2nd antibody: anti Rabbit 1:10000
5% BSA blocking
Exposure time 30s
Total protein, Stained by reversible protein stain kit, Thermo, 24585
Ladder: BioRad Cat# 1610395
Total protein
kDa
250
150
100
gamma H2A.X
75
50
37
25
20
Con
SS-31
DOX
DOX+SS-31
15
10
gamma H2A.X
15 kDa

## Slide 9
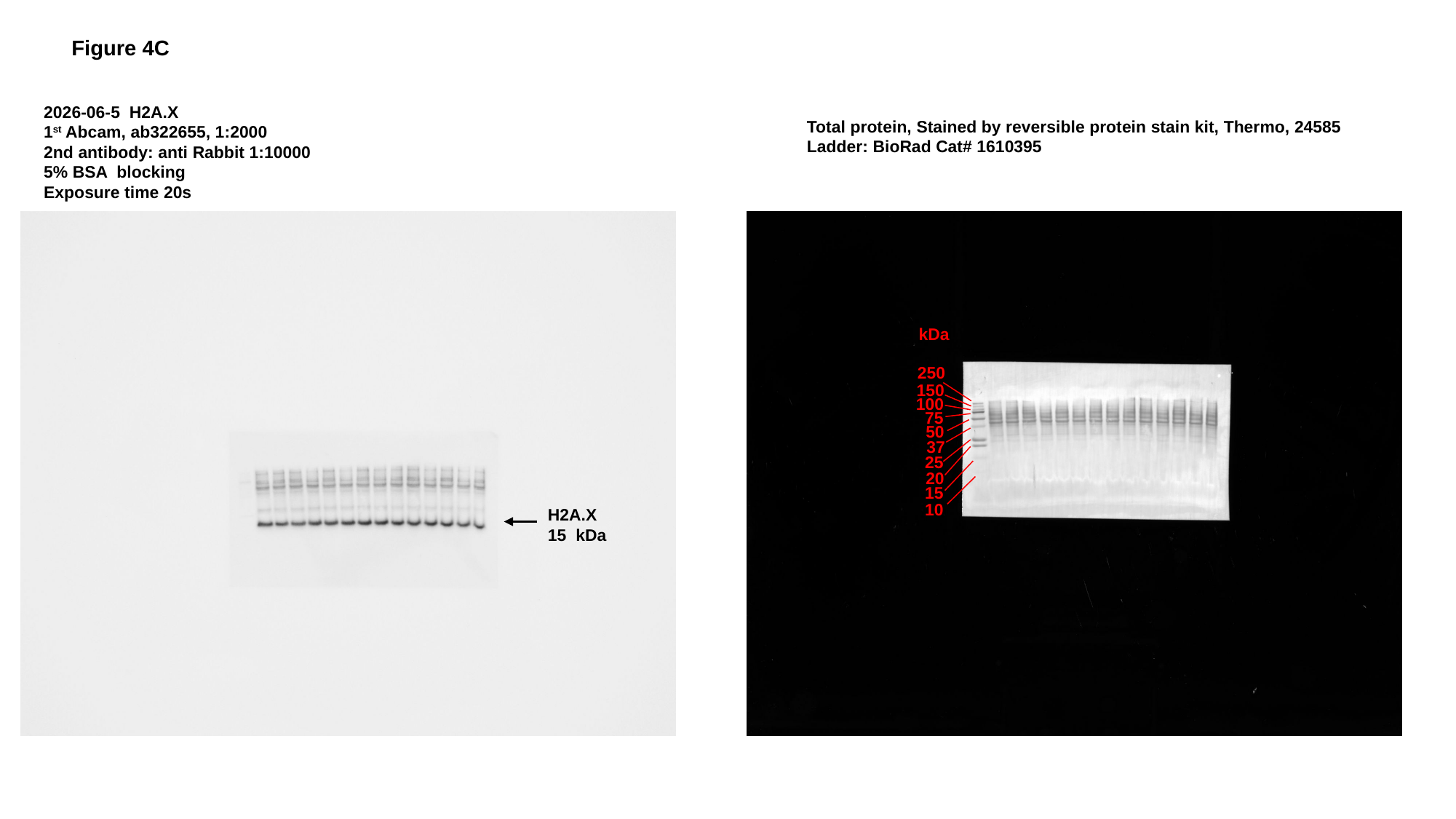

Figure 4C
2026-06-5 H2A.X
1st Abcam, ab322655, 1:2000
2nd antibody: anti Rabbit 1:10000
5% BSA blocking
Exposure time 20s
Total protein, Stained by reversible protein stain kit, Thermo, 24585
Ladder: BioRad Cat# 1610395
kDa
250
150
100
75
50
37
25
20
15
10
H2A.X
15 kDa

## Slide 10
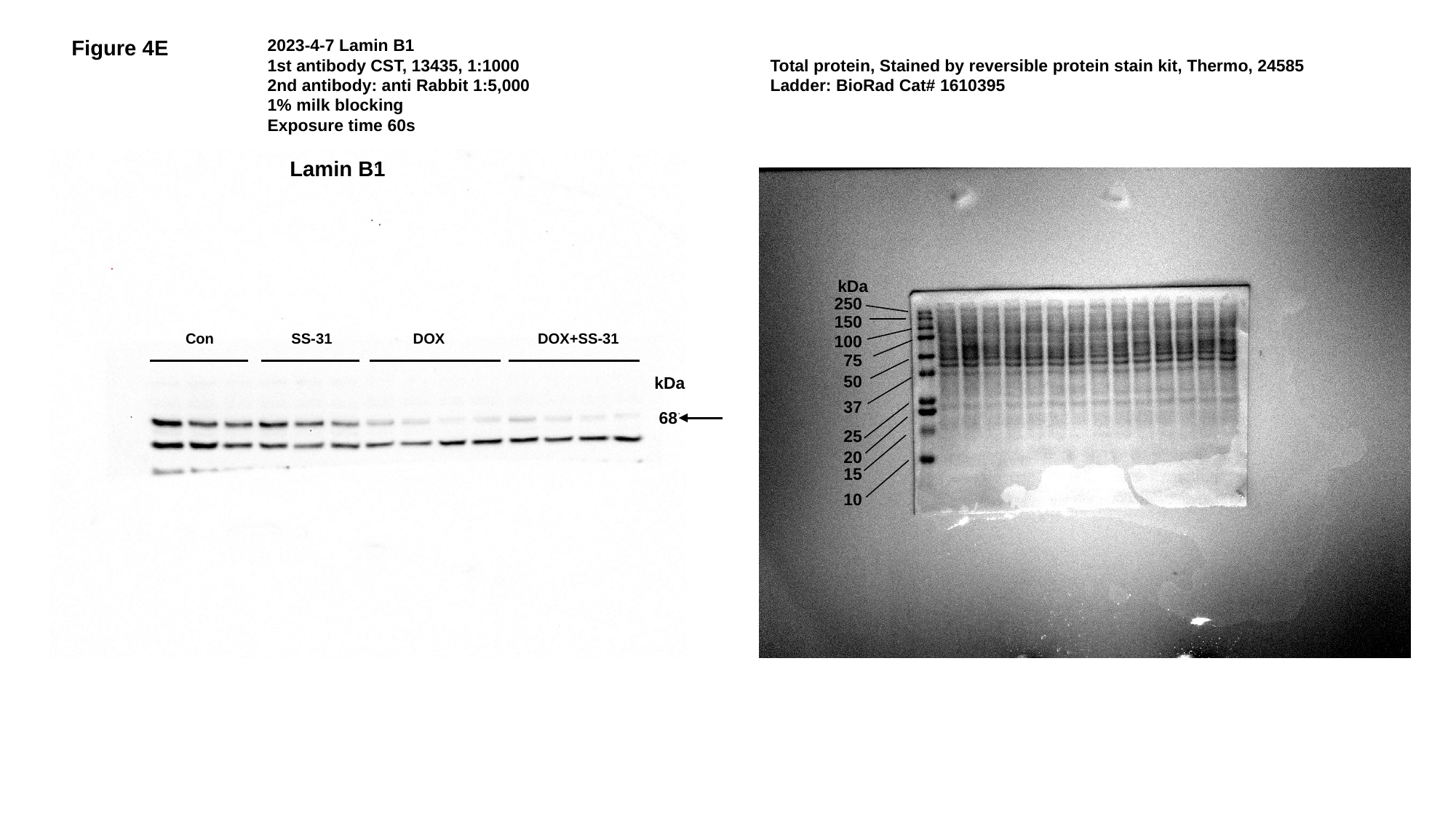

Figure 4E
2023-4-7 Lamin B1
1st antibody CST, 13435, 1:1000
2nd antibody: anti Rabbit 1:5,000
1% milk blocking
Exposure time 60s
Total protein, Stained by reversible protein stain kit, Thermo, 24585
Ladder: BioRad Cat# 1610395
Lamin B1
kDa
250
150
100
Con
SS-31
DOX
DOX+SS-31
75
50
kDa
37
68
25
20
15
10

## Slide 11
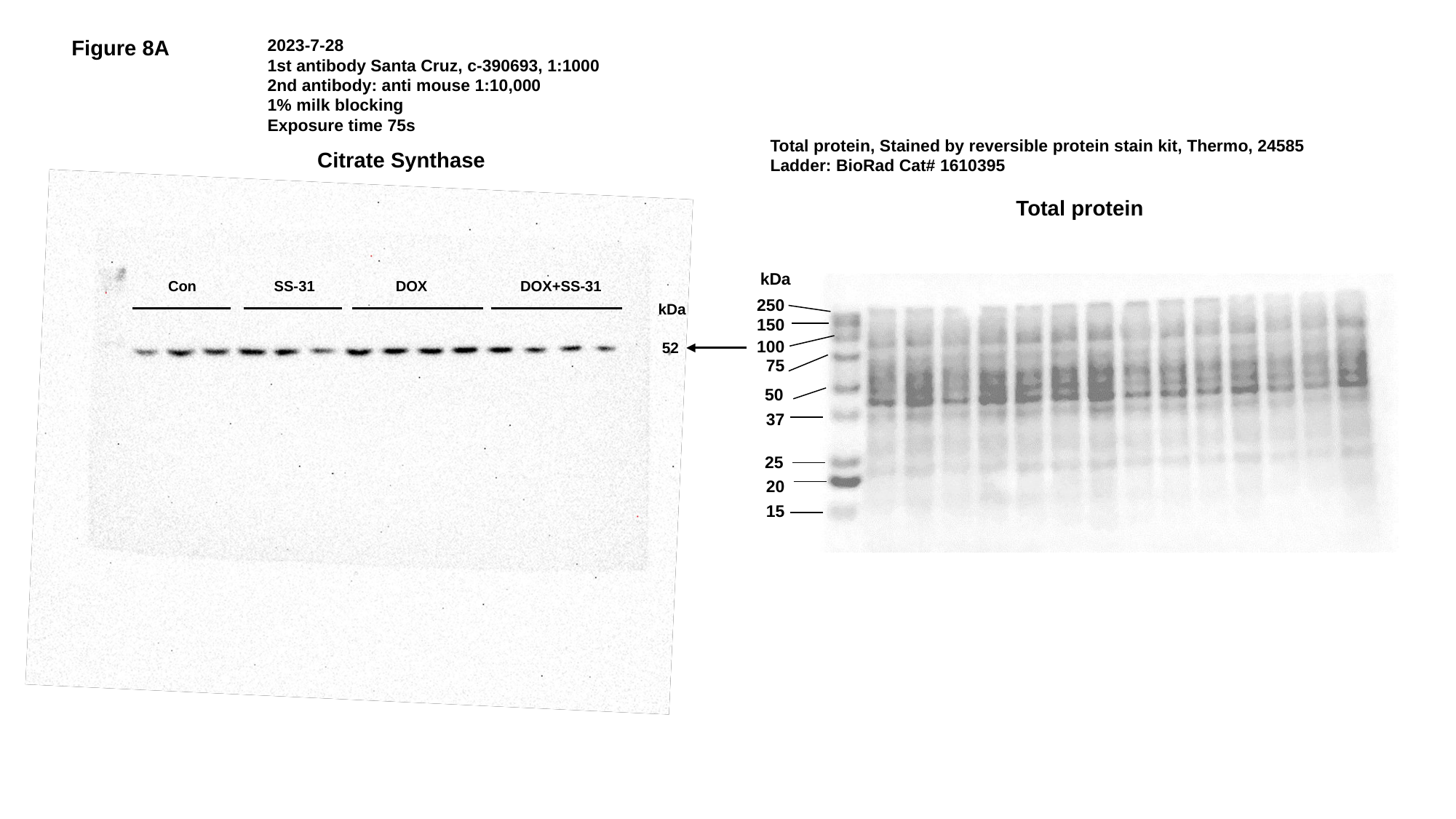

Figure 8A
2023-7-28
1st antibody Santa Cruz, c-390693, 1:1000
2nd antibody: anti mouse 1:10,000
1% milk blocking
Exposure time 75s
Total protein, Stained by reversible protein stain kit, Thermo, 24585
Ladder: BioRad Cat# 1610395
Citrate Synthase
Total protein
kDa
Con
SS-31
DOX
DOX+SS-31
250
kDa
150
100
52
75
50
37
25
20
15

## Slide 12
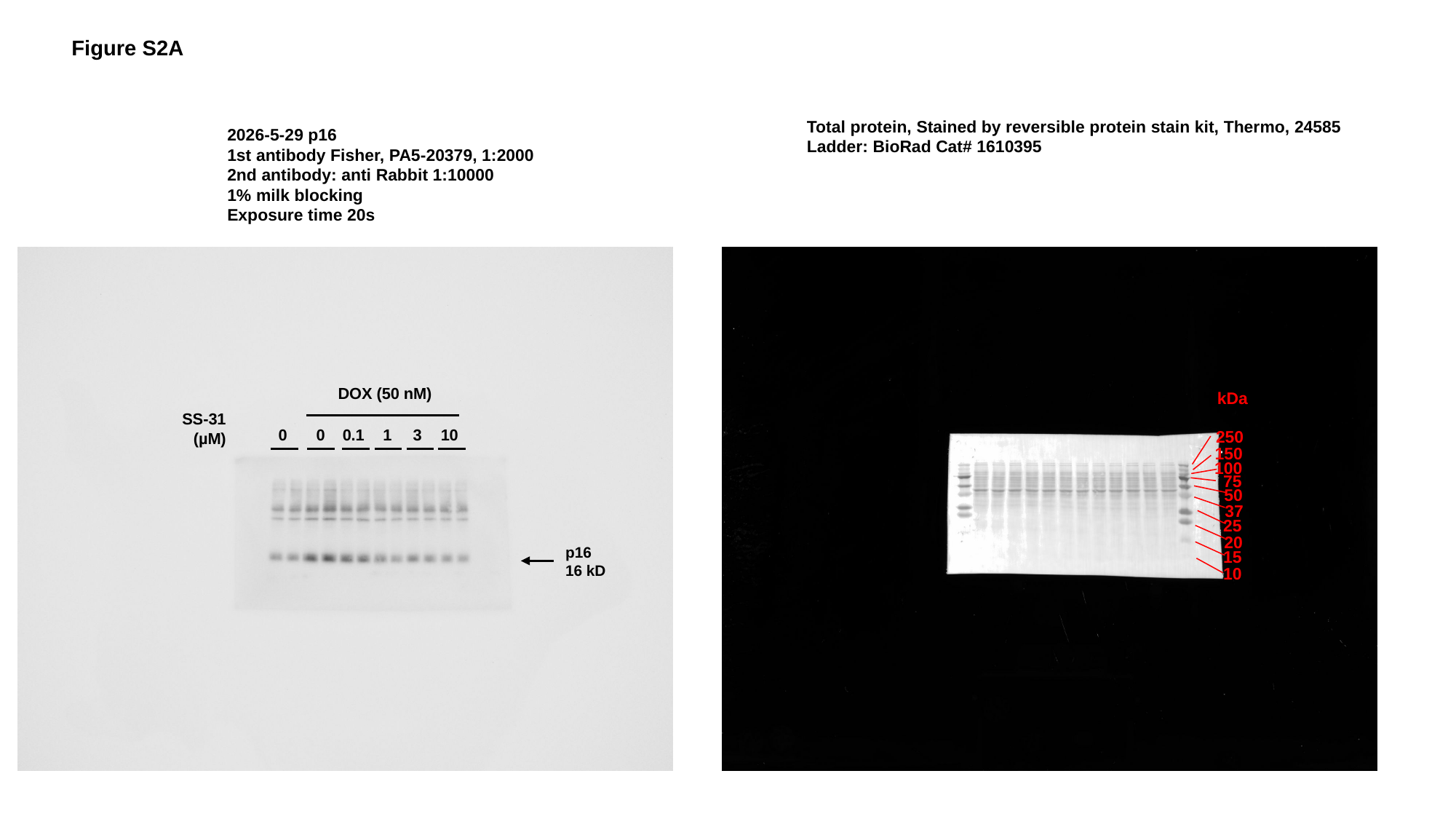

Figure S2A
Total protein, Stained by reversible protein stain kit, Thermo, 24585
Ladder: BioRad Cat# 1610395
2026-5-29 p16
1st antibody Fisher, PA5-20379, 1:2000
2nd antibody: anti Rabbit 1:10000
1% milk blocking
Exposure time 20s
DOX (50 nM)
kDa
SS-31 (µM)
250
0
0
0.1
1
3
10
150
100
75
50
37
25
20
p16
16 kD
15
10

## Slide 13
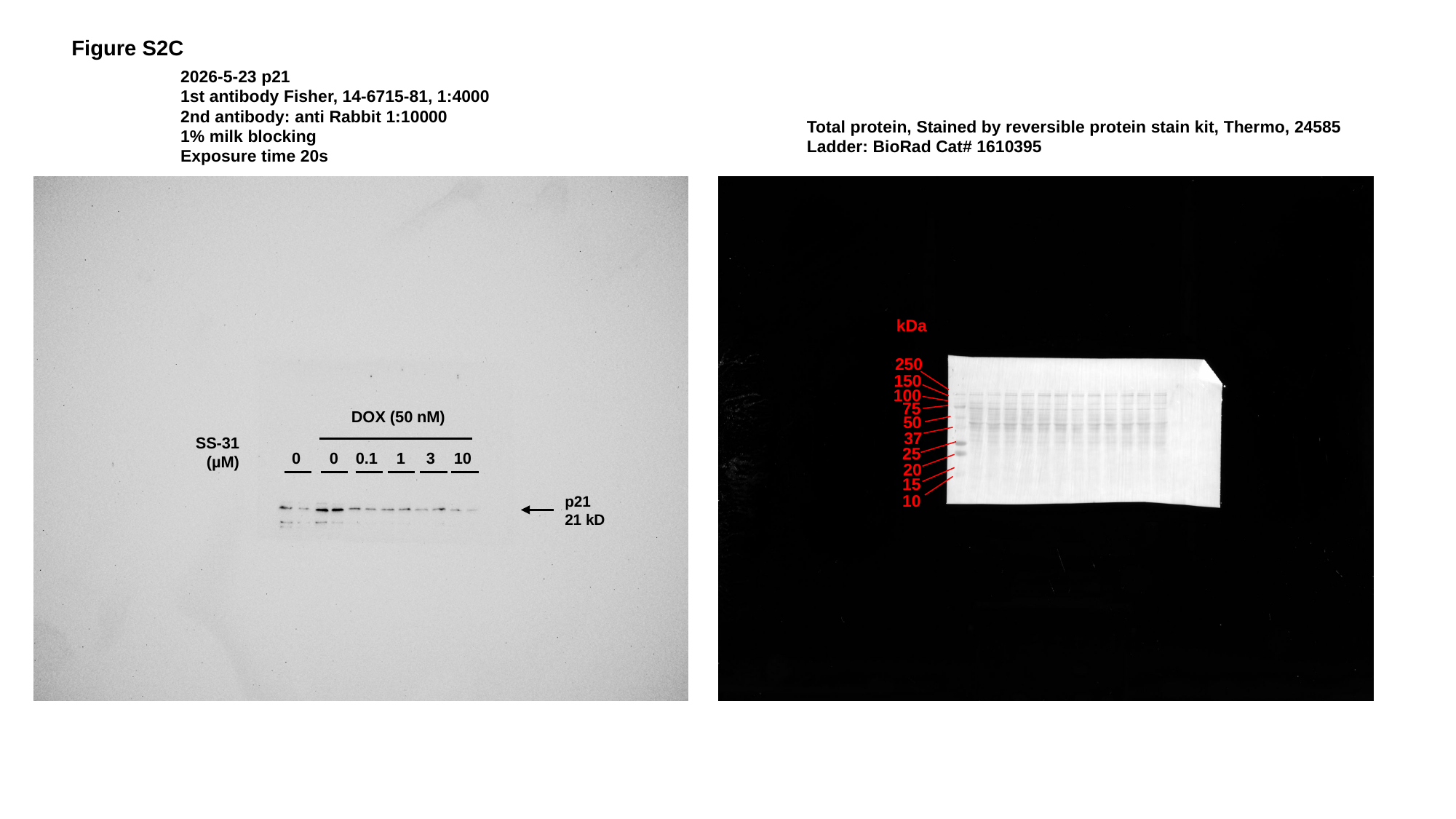

Figure S2C
2026-5-23 p21
1st antibody Fisher, 14-6715-81, 1:4000
2nd antibody: anti Rabbit 1:10000
1% milk blocking
Exposure time 20s
Total protein, Stained by reversible protein stain kit, Thermo, 24585
Ladder: BioRad Cat# 1610395
kDa
250
150
100
75
DOX (50 nM)
50
37
SS-31 (µM)
25
0
0
0.1
1
3
10
20
15
10
p21
21 kD

## Slide 14
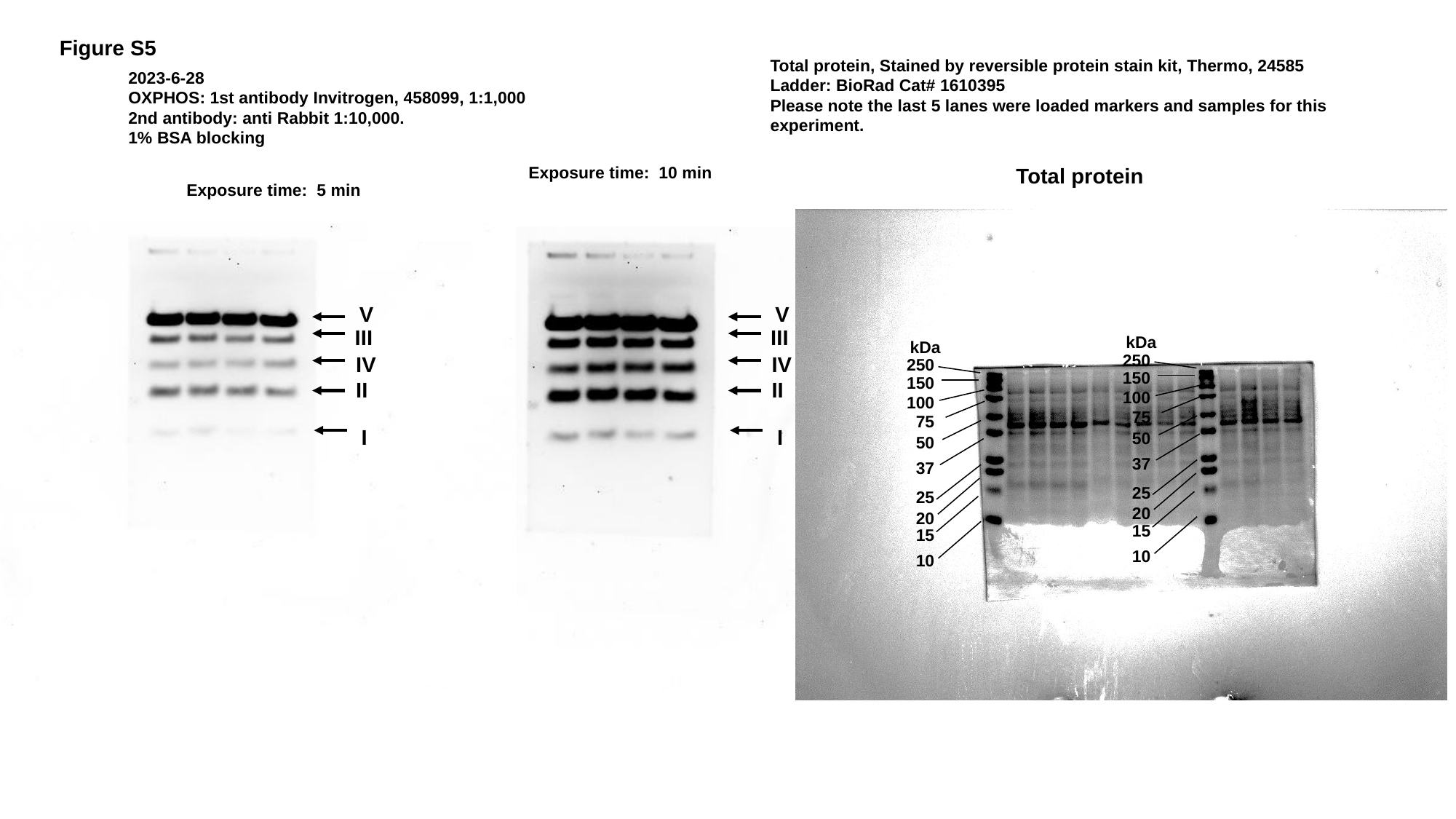

Figure S5
Total protein, Stained by reversible protein stain kit, Thermo, 24585
Ladder: BioRad Cat# 1610395
Please note the last 5 lanes were loaded markers and samples for this experiment.
2023-6-28
OXPHOS: 1st antibody Invitrogen, 458099, 1:1,000
2nd antibody: anti Rabbit 1:10,000.
1% BSA blocking
Exposure time:  10 min
Total protein
Exposure time:  5 min
V
V
III
III
kDa
kDa
250
IV
IV
250
150
150
II
II
100
100
75
75
I
I
50
50
37
37
25
25
20
20
15
15
10
10
