# Supplementary material for: Mitochondrial-Targeted SS-31 Attenuates the Doxorubicin-Induced Cardiomyoblast H9C2 Cell Senescence
Source: Biology (Basel). 2026 Jun 28;15(13):1034. doi: 10.3390/biology15131034 (PMC13359912; doi:10.3390/biology15131034)
Supplement: Supplementary file 1 [file biology-15-01034-s001.zip › biology-4322693-supplementary.pdf]

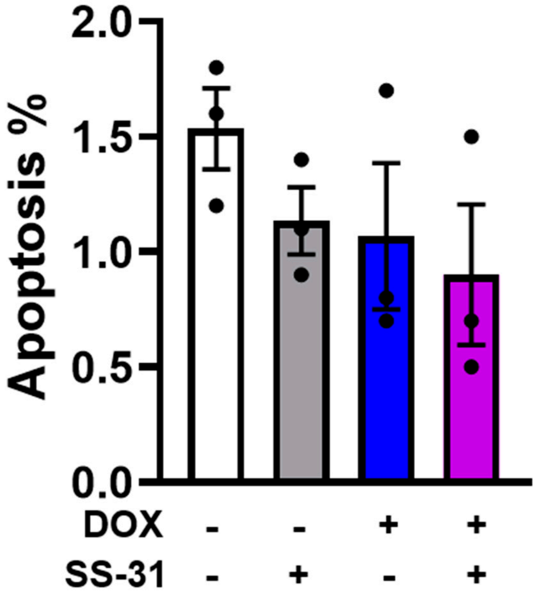

Figure S1. 50 nM DOX did not induce cell apoptosis. N =3 independent experiments. All the data are mean ± SEM.

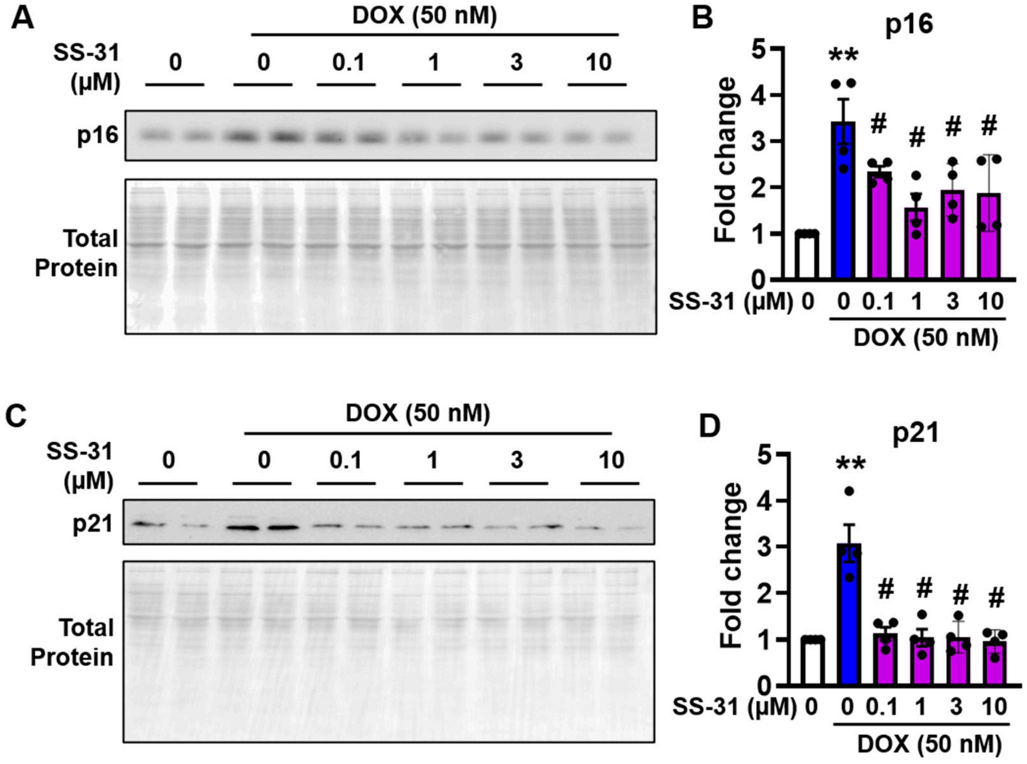

Figure S2. SS-31 dose effect on the DOX induced elevation of p16 and p21. (A, C) Representative Western blot images of p16 and p21. (B) quantification of the p16 and p21 expression level normalized to control. N = 4 in each group. All the data are mean ± SEM.

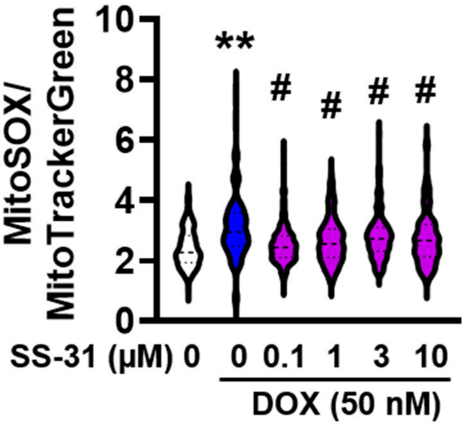

Figure S3. SS-31 dose effect on the DOX induced elevation of mitochondrial superoxide production. N = 152-186 cells from 3 independent preparations. All the data are mean ± SEM.

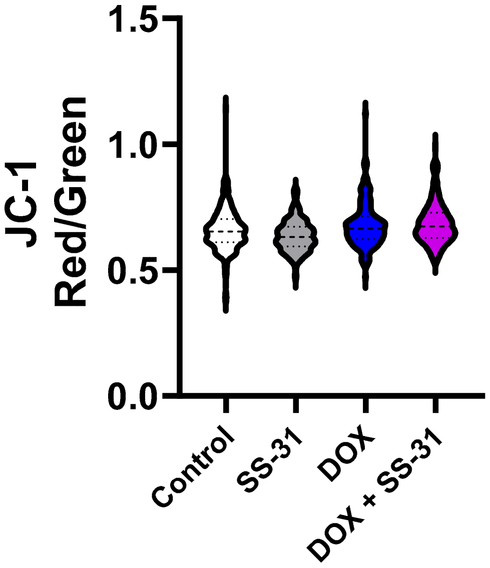

Figure S4. 50 nM DOX did not decrease the mitochondrial membrane potential. N = 170-197 cells from 3 independent preparations. All the data are mean ± SEM.

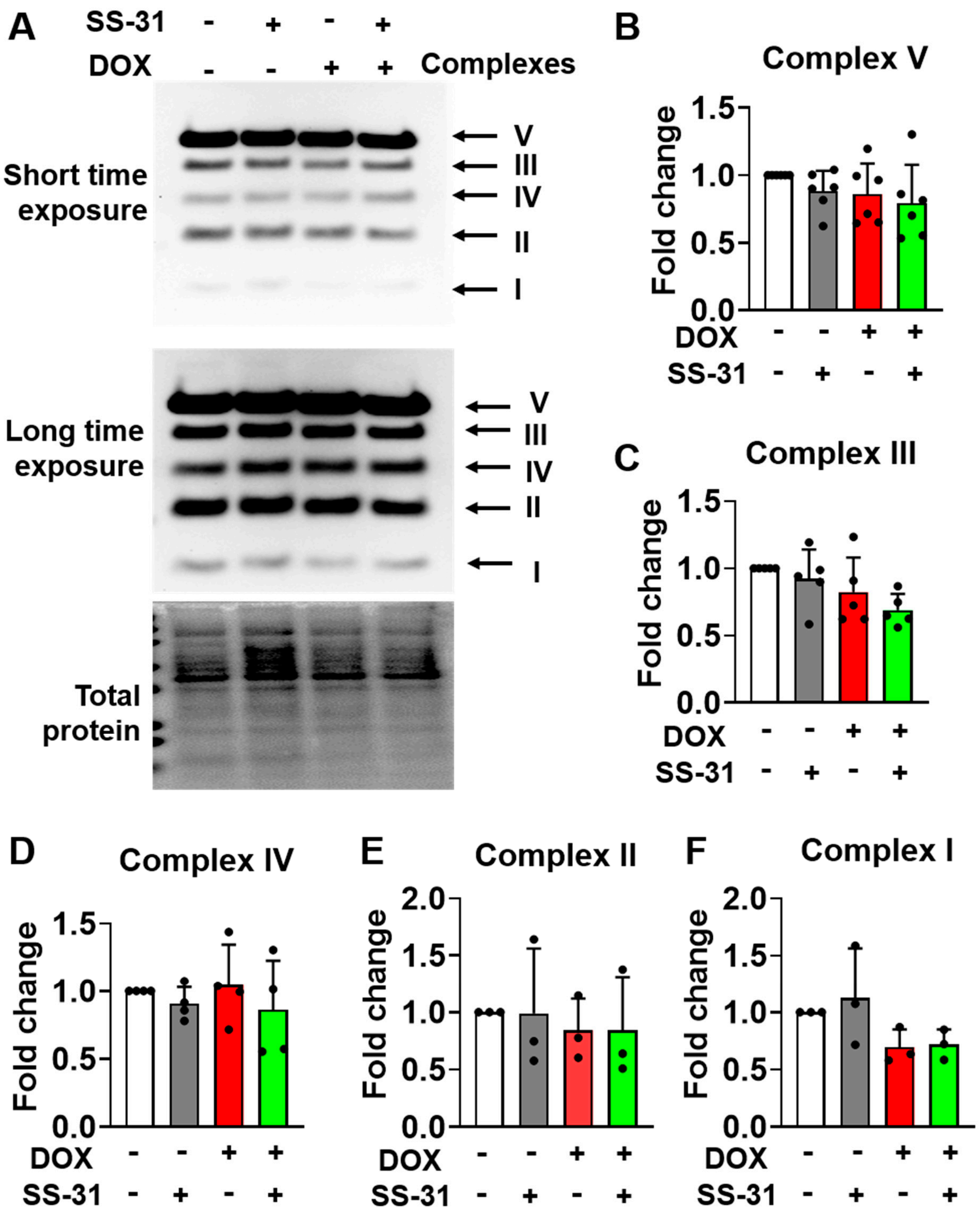

Figure S5. 50 nM DOX does not alter the protein abundance of mitochondrial respiratory complexes. (A) Representative Western blot images showing the expression levels of the five mitochondrial respiratory complexes in H9C2 cells 3 days after a 3-hour exposure to 50 nM DOX. (B-F) the quantification of the individual complex subunits. No significant differences were observed in the protein abundance of any respiratory complex between the control and DOX treated groups. N = 4-5 independent experiments. All the data are mean ± SEM.
